# Deconer: An Evaluation Toolkit for Reference-based Deconvolution Methods Using Gene Expression Data

**DOI:** 10.1093/gpbjnl/qzaf009

**Published:** 2025-02-18

**Authors:** Wei Zhang, Xianglin Zhang, Qiao Liu, Lei Wei, Xu Qiao, Rui Gao, Zhiping Liu, Xiaowo Wang

**Affiliations:** Center of Intelligent Medicine, School of Control Science and Engineering, Shandong University, Jinan 250061, China; Department of Clinical Laboratory, The Second Hospital, Cheeloo College of Medicine, Shandong University, Jinan 250033, China; Department of Statistics, Stanford University, Stanford, CA 94305, USA; Ministry of Education Key Laboratory of Bioinformatics; Center for Synthetic and Systems Biology; Bioinformatics Division, Beijing National Research Center for Information Science and Technology; Department of Automation, Tsinghua University, Beijing 100084, China; Center of Intelligent Medicine, School of Control Science and Engineering, Shandong University, Jinan 250061, China; Center of Intelligent Medicine, School of Control Science and Engineering, Shandong University, Jinan 250061, China; Center of Intelligent Medicine, School of Control Science and Engineering, Shandong University, Jinan 250061, China; Ministry of Education Key Laboratory of Bioinformatics; Center for Synthetic and Systems Biology; Bioinformatics Division, Beijing National Research Center for Information Science and Technology; Department of Automation, Tsinghua University, Beijing 100084, China

**Keywords:** Deconer, Deconvolution benchmark, Gene expression, Cell-type proportion, Reference-based deconvolution

## Abstract

In recent years, computational methods for quantifying cell-type proportions from transcription data have gained significant attention, particularly those reference-based methods which have demonstrated high accuracy. However, there is currently a lack of comprehensive evaluation and guidance for available reference-based deconvolution methods in cell-type deconvolution analysis. In this study, we introduce Deconvolution Evaluator (Deconer), a comprehensive toolkit for the evaluation of reference-based deconvolution methods. Deconer provides various simulated and real gene expression datasets, including both bulk and single-cell sequencing data, and offers multiple visualization interfaces. By utilizing Deconer, we conducted systematic comparisons of 16 reference-based deconvolution methods from different perspectives, including method robustness, accuracy in deconvolving rare components, signature gene selection performance, and external reference construction capability. We also performed an in-depth analysis of the application scenarios and challenges in cell-type deconvolution methods. Finally, we provided constructive suggestions for users to select and develop cell-type deconvolution algorithms. This study provides novel insights for researchers, assisting them in choosing appropriate toolkits, applying solutions in clinical contexts, and advancing the development of deconvolution tools tailored to gene expression data. The tutorials, manual, source code, and demo data of Deconer are publicly available at https://honchkrow.github.io/Deconer/ and https://ngdc.cncb.ac.cn/biocode/tool/7577.

## Graphical abstract

**Figure:**
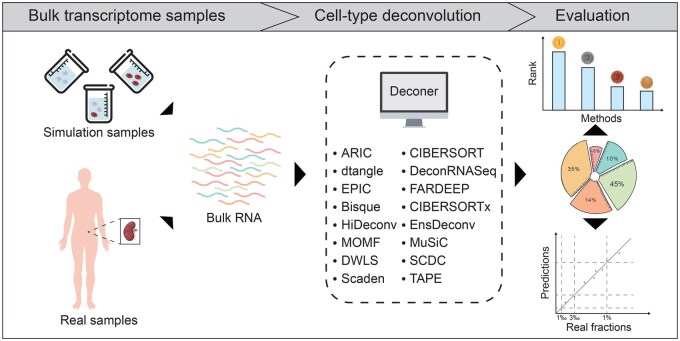

## Introduction

The rapid development of high-throughput sequencing technologies, such as RNA sequencing (RNA-seq) and single-cell RNA-seq (scRNA-seq), provides us unprecedented opportunities to unravel the global gene expression in cells [1,2]. In multicellular organisms, nearly all physiological and pathological processes involve multiple types of cells, each playing a unique role in specific mechanisms [3,4]. Quantifying cell-type proportions is essential for tracking signals associated with certain phenotypes or diseases. For example, predicting cell-type proportion aids in uncovering the cellular composition and heterogeneity of tissues and organs, providing vital information for studying cell systems [5]. Estimation of cell-type proportions helps researchers understand the infiltration of immune cells in the tumor microenvironment, which is crucial for selecting appropriate immunotherapy strategies [6]. Additionally, by analyzing the proportion of cell types in tissue samples, we can enhance the accuracy of disease diagnosis and determine the stage of disease progression [7,8].

So far, the proportions of different cell types can be determined through various experimental methods based on physical properties, such as flow cytometry, immunohistochemistry, and fluorescence-activated cell sorting (FACS) [9,10]. However, these techniques have limitations in certain aspects. For instance, flow cytometry is the gold standard for identification of cell subsets, but its throughput might be relatively low [11–13]. Furthermore, FACS relies on the availability of suitable cell surface markers. In some cases, the lack of specific markers may result in unfeasibility to effectively capture and identify particular cell types [12,14]. The advance of single-cell sequencing technologies has revolutionized our understanding of cellular heterogeneity and allowed us to probe the functional role of each cell type within complex multicellular systems. However, a considerable number of bulk samples are still generated in clinical and experimental research, and numerous bulk gene expression data in existing databases remain underutilized and untapped. Therefore, computational models for quantifying cell-type proportions remain highly relevant and essential.

The proportions of different cell types can be inferred from various types of omics data, including gene expression data, DNA methylation data, and DNA accessibility data [15–18]. Currently, DNA accessibility data have been utilized to infer cell-type proportions in some studies, but there is limited research in this regard. DNA methylation data are one of the most commonly used data types for this task. While providing valuable insights into gene regulation, this type of data involves more complex experimental and data-processing procedures, which indicates that it is more suitable for assisting in cell-type proportion prediction when analyzing transcriptional regulation. Of course, in certain situations, DNA methylation data may be more appropriate for deciphering cell-type proportions, such as predicting the circulating tumor DNA burden in cell-free DNA samples [7,19,20]. Gene expression data, on the other hand, directly reflect transcriptional differences among various cell types. These differences can serve as features for identifying different cell-type proportions. Furthermore, gene expression data have a direct connection to cellular functions and biological processes, making cell-type deconvolution results more biologically meaningful. Compared to the previously mentioned data types, gene expression data can be more easily obtained experimentally and can be processed using standard normalization methods, such as fragments per kilobase of transcript per million mapped reads (FPKM) and transcripts per million (TPM).

Cell-type deconvolution algorithms for gene expression data can be primarily divided into two categories: reference-based methods and reference-free methods. Reference-based methods utilize pre-defined cell-type-specific gene expression profiles as prior information and apply techniques, such as constrained linear regression, to estimate cell-type proportions. In contrast, reference-free methods do not rely on external expression references and directly obtain cell-type proportions and expression profiles using techniques such as non-negative matrix factorization (NMF). Reference-free methods need to simultaneously estimate expression profiles and cell-type proportions, which might attenuate the deconvolution performance in comparison with reference-based methods [18,21–24]. Hence, it is crucial to evaluate and compare these reference-based deconvolution methods for swiftly and accurately deciphering the proportions of distinct cell types.

**Figure 1:**
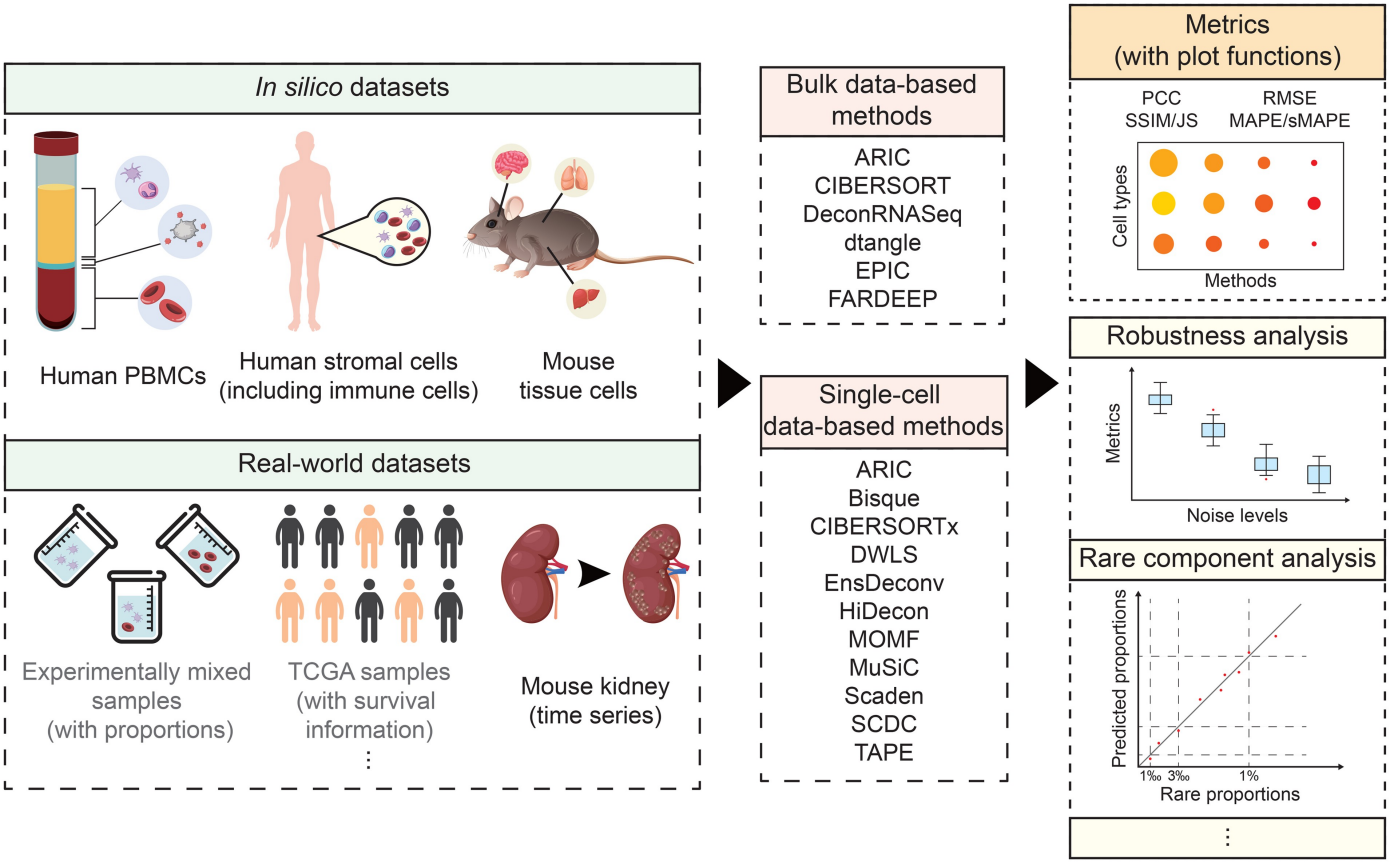
**Overview workflow of this study and the main functions in Deconer**The left panel shows the datasets provided by Deconer. The data marked in black font were utilized in this study, while the data displayed in gray font were pre-processed but not used in the comparison analysis conducted in this study. The middle panel lists all the deconvolution methods evaluated. Some main functions provided by Deconer are shown in the right panel. Deconer, Deconvolution Evaluator; PBMC, peripheral blood mononuclear cell; TCGA, The Cancer Genome Atlas; CIBERSORT, Cell-type Identification By Estimating Relative Subsets Of RNA Transcripts; EPIC, Estimating the Proportion of Immune and Cancer cells; FARDEEP, Fast And Robust DEconvolution of Expression Profiles; DWLS, dampened weighted least squares; EnsDeconv, Ensemble Deconvolution; HiDecon, Hierarchical Deconvolution; MOMF, Multi-Omics Matrix Factorization; MuSiC, MUlti-Subject SIngle Cell deconvolution; Scaden, single cell-assisted deconvolutional deep neural network; TAPE, Tissue-AdaPtive autoEncoder; PCC, Pearson correlation coefficient; RMSE, root mean square error; MAPE, mean absolute percentage error; sMAPE, symmetric mean absolute percentage error; SSIM, structural similarity index measure; JS, Jensen–Shannon divergence.

In this study, we developed a specialized tool named Deconvolution Evaluator (Deconer) (**Figure 1**) to streamline systematic comparisons. Inspired by the Dialogue for Reverse Engineering Assessment and Methods (DREAM) Challenge [25], Deconer enables a more comprehensive performance evaluation across different deconvolution methods. First, we conducted an extensive evaluation analysis of 16 reference-based deconvolution algorithms, including 14 conventional deconvolution methods based on probabilistic or machine learning models, and 2 recently proposed deep learning-based methods which have garnered significant attention (**Table 1**). Second, we examined various factors that impact deconvolution processes, such as data noise, the number of cellular components, and the presence of rare cell types. Besides, Deconer incorporates several simulation data generation techniques using both bulk and single-cell gene expression data. We also compiled a large set of high-quality RNA-seq and scRNA-seq datasets, some of which were experimentally derived with known cell-type proportions, providing a valuable resource for method comparison. Furthermore, multiple evaluation metrics were used for analyzing and interpreting the results, offering insights into selecting the most suitable deconvolution methods for different research scenarios. All assessments in this study were performed using Deconer, allowing users to reproduce the results and facilitating future comparisons and evaluations of related deconvolution methods. We have made Deconer and the processed datasets publicly available, allowing for easier result reproduction and comparison of new deconvolution algorithms.

**Table 1:** The deconvolution algorithms for gene expression data involved in this study

Deconvolution method	Reference data type	Description and remark
ARIC	Bulk & single-cell	ARIC adopts component-wise condition number and can be used for multiple data types; implemented as a Python package (v0.1.0)
Bisque	Single-cell	NNLS regression is used for estimating cell proportions; implemented as an R package (v1.0.5)
CIBERSORT*	Bulk	A well-known deconvolution method adopts linear ν-SVR
CIBERSORTx	Single-cell	The updated version of CIBERSORT with user-friendly web interface
DeconRNASeq	Bulk	One of the earliest deconvolution methods using constrained linear regression model; implemented as an R package (v1.40.0)
dtangle	Bulk	A newly proposed method robust to outliers; implemented as an R package (v2.0.9)
DWLS	Single-cell	DWLS adopts dampened weighted least squares and can correct common biases towards cell types; implemented as an R package (v0.1.0)
EnsDeconv	Single-cell	EnsDeconv employs an ensemble learning approach to enhance model stability; implemented as an R package (v0.2.1)
EPIC	Bulk	EPIC is robust to unknown mixture content; implemented as an R package (v1.1.6)
FARDEEP	Bulk	FARDEEP adopts least trimmed squares and is robust to outliers; implemented as an R package (v1.0.1)
HiDecon	Single-cell	HiDecon uses hierarchical deconvolution method; implemented as an R package (v0.3.2)
MOMF	Single-cell	MOMF is based on Poisson distribution and alternating direction method of multipliers; implemented as an R package (v0.2.0)
MuSiC	Single-cell	MuSiC assigns weights to genes to maintain cross-subject and cross-cell consistency; implemented as an R package (v1.0.0)
Scaden	Single-cell	The first proposed deep learning-based deconvolution algorithm; implemented as a Python package (v1.1.2)
SCDC	Single-cell	SCDC adopts an ENSEMBLE strategy for addressing the problem of batch effect confounding; implemented as an R package (v0.0.0.9000)
TAPE	Single-cell	A method based on autoencoder architecture; implemented as a Python package (v1.1.2)

*Note*: *, the version implemented in EpiDISH package (v2.14.1) was used since the website of CIBERSORT is archived. NNLS, non-negative least squares; SVR, support vector regression; CIBERSORT, Cell-type Identification By Estimating Relative Subsets Of RNA Transcripts; EPIC, Estimating the Proportion of Immune and Cancer cells; FARDEEP, Fast And Robust DEconvolution of Expression Profiles; DWLS, dampened weighted least squares; EnsDeconv, Ensemble Deconvolution; HiDecon, Hierarchical Deconvolution; MOMF, Multi-Omics Matrix Factorization; MuSiC, MUlti-Subject SIngle Cell deconvolution; Scaden, single cell–assisted deconvolutional deep neural network; TAPE, Tissue-AdaPtive autoEncoder.

## Method

### Toolkit

We developed Deconer, a comprehensive evaluation toolkit for cell-type deconvolution algorithms, as a free R package. The main functions of Deconer are displayed in Figure 1. Deconer includes a variety of functions for performance assessment. The aforementioned pseudo-bulk data can be directly generated through Deconer. Deconer also offers a diverse range of pre-processed real experimental datasets with known cell-type proportions [26–33]. A gene expression dataset for experimental simulation of chronic kidney disease in mouse kidney is also provided. In addition, gene expression profiles for 32 cancer types from The Cancer Genome Atlas (TCGA) and corresponding clinical survival data are also integrated [34–36]. Deconer integrates a variety of evaluation metrics and plotting tools. Furthermore, it offers several evaluation functions, such as stability testing of the model under simulated noise conditions and accuracy analysis of rare component deconvolution.

### Overview of reference-based cell-type deconvolution algorithms for gene expression data

Reference-based deconvolution methods can be mainly classified into two subcategories: those using bulk data as a reference and those using single-cell data as a reference. Typical methods employing bulk data as a reference include Tumor IMmune Estimation Resource (TIMER) [37], Digital Sorting Algorithm (DSA) [38], xCell [39], Microenvironment Cell Populations-counter (MCP-counter) [28], DeconRNASeq [40], Estimating the Proportion of Immune and Cancer cells (EPIC) [41], dtangle [3], Fast And Robust DEconvolution of Expression Profiles (FARDEEP) [42], and Cell-type Identification By Estimating Relative Subsets Of RNA Transcripts (CIBERSORT) [43], while those utilizing single-cell data as a reference encompass Bisque [44], CIBERSORTx [10], dampened weighted least squares (DWLS) [45], Multi-Omics Matrix Factorization (MOMF) [46], MUlti-Subject SIngle Cell deconvolution (MuSiC) [47], single cell-assisted deconvolutional deep neural network (Scaden) [48], Tissue-AdaPtive autoEncoder (TAPE) [49], Hierarchical Deconvolution (HiDecon) [50], Ensemble Deconvolution (EnsDeconv) [51], and SCDC [52]. Notably, both bulk and single-cell data can serve as an external reference for ARIC [4]. We conducted a systematic review of these methods and selected 16 methods for comparison in this study (File S1). Other methods that have already been compared and analyzed in previous studies are not included in our current analysis [18,23,41]. Among these methods, the majority are based on the linear regression model, where the measured sample gene expression values are the sum of gene expression values from different types of cells. Although these linear regression-based methods share a common conceptual foundation, they differ in focal points and approaches to proportional estimation. For example, DeconRNASeq adopts quadratic programming for estimating the mixing proportions of distinctive cell types while Bisque utilizes non-negative least squares (NNLS) [40]. EPIC aims to achieve precise differentiation and proportion estimation of distinct cell types by seeking out genes with highly specific expression [41]. dtangle uses a biologically appropriate linear mixing model and uses log-transformed data to fit the model [3]. CIBERSORT estimates relative subsets of RNA transcripts using support vector regression (SVR), and CIBERSORTx is an updated version for single-cell data [10,43]. MOMF directly models gene count data using a Poisson distribution and employs the alternating direction method of multipliers (ADMM) approach to reduce computational load [46]. FARDEEP, ARIC, DWLS, and MuSiC achieve auto-selection or weighting of different signature genes by analyzing the expression profiles of various cell types and employ approaches such as adaptive deconvolution to estimate proportions accurately [4,42,45,47]. HiDecon uses a hierarchical deconvolution method [50]. EnsDeconv and SCDC, on the other hand, employ an ensemble learning approach, integrating multiple datasets for deconvolution to enhance model stability [51,52]. It is worth mentioning that this study incorporates two recently proposed deconvolution methods based on deep learning techniques, namely Scaden and TAPE. Scaden is the first deconvolution method to employ deep learning techniques [48]. It uses a multi-layer fully connected neural network that directly takes high-variance genes as input to estimate proportions of different cell types. On the other hand, TAPE utilizes an autoencoder architecture, simultaneously estimating both cell-type proportions and gene expression profiles [49]. All the aforementioned methods and their corresponding descriptions are summarized in Table 1.

### Evaluation datasets

In this study, we extended the previously collected data from our previous work with additional bulk RNA-seq and scRNA-seq datasets [4]. In total, we provided four types of pseudo-bulk data: two were generated through simulation of bulk RNA-seq data, while the other two were generated through simulation of scRNA-seq data. Inspired by the tumor deconvolution DREAM challenge, we collected 233 high-quality bulk RNA-seq samples to simulate cell populations, including stromal cells and immune cells. We constructed two distinct scenarios, corresponding to “coarse” and “fine” conditions, respectively. Under the coarse condition, mixed samples contained 8 different cell types, whereas under the fine condition, mixed samples contained 14 different cell types. Additionally, we collected a unique single-cell dataset consisting of 7 different tissues from fetal mice [53]. In this dataset, each cell type has 1500 single-cell data entries, which ensures adequate data for the construction of simulated samples and external references. Furthermore, given the relatively large expression profile differences among these 7 tissues, samples generated using this dataset can provide a fair performance evaluation for various algorithms [53]. We also collected 10K human peripheral blood mononuclear cell (PBMC) scRNA-seq data. For this dataset, we employed multimodal omics analysis (MUON) to annotate 13 reliable cell types of 11,094 cells (Figure S1) [54].

In addition to the simulation data, we also employed a well-characterized mouse kidney dataset to test the comparison methods [34,35]. This dataset represents a real-world case with unknown proportions, and we assessed the accuracy of the derived cell-type proportions by associating them with known biological phenotypes or processes.

We adopted different approaches for generating evaluation data from bulk or single-cell gene expression data. For bulk data, we first divided all samples into training (70%) and test sets (30%). The training sets were solely used to generate the external reference, while the test sets were utilized to produce samples for assessment. Evaluation samples were directly generated using the same linear mixing approach as in a previous study [4]. In brief, numerical linear combinations were performed after generating cell-type proportions according to the requirements of different evaluation criteria. As for scRNA-seq data, we initially divided the cells for each cell type into a training set (70%) and a test set (30%) based on the number of cells. The training set was also only used for generating an external reference. To generate pseudo-bulk data, we first created random cell-type proportions and then sampled cells from the test set based on the required cell number. Then, the gene expression values of the cells were added together to simulate the bulk sample. More detailed descriptions of the data generation methods under different scenarios are provided in each section.

All the collected data are summarized in **Table 2**, Table S1, and File S1. The accession numbers are summarized in Table S2. The pre-processing workflow for all datasets is provided in File S1. The process of signature gene selection is detailed in File S1. All datasets are provided on the Deconer website (https://honchkrow.github.io/Deconer_dataset/).

**Table 2:** Summary of the test datasets used in this study

Dataset	Number of cell types	Data type	Description
coarse_bulk	8	Bulk	This dataset is derived from 233 high-quality bulk RNA-seq samples to simulate stromal cell populations, including immune cell populations; for each cell type, RNA-seq samples are collected from at least two distinct studies
fine_bulk	14	Bulk	This dataset, like the coarse_bulk dataset, has data for all cell types derived from at least two different datasets, except for regulatory T cells which are only sourced from a single dataset
mouse_tissue	7	Single-cell	This dataset contains 7 well-characterized mouse tissues
human_PBMC	13	Single-cell	10K human PBMC scRNA-seq data from 10X
mouse_kidney	13	Single-cell	This dataset is derived from UUO mouse model of renal fibrosis in kidney diseases

*Note*: Deconer provides all datasets listed in this table, along with additional datasets with known cell-type proportions. For a detailed summary table, please visit https://honchkrow.github.io/Deconer_dataset/. RNA-seq, RNA sequencing; scRNA-seq, single-cell RNA sequencing; PBMC, peripheral blood mononuclear cell; UUO, unilateral ureteric obstruction.

### Simulation of data noise for gene expression data

According to the study by Jin et al., the negative binomial model was found to be highly effective in capturing salient features in real data, such as mean–variance trends and sample–sample concordance [18]. The model offered a depiction of the noise structure intrinsic to real data, thereby enabling more accurate interpretation and analysis. Inspired by these findings, we adopted the negative binomial model to simulate various levels of noise as the previous work [18,55]. In brief, the model can be described as follows:


$$\begin{array}{c} \mu_{i0}=r_{i0}\times L_{j}\;\\ \begin{array}{c} \sigma_{i}=\left(1.8\times p_{t}+\frac{1}{\sqrt{\mu_{i0}}}\right)\times e^{\frac{\delta }{2}}\;\\ \begin{array}{c} \mu_{ij}=\mathrm{Gamma}\left(\mathrm{shape}=\frac{1}{{\sigma_{i}}^{2}},\;\mathrm{scale}=\frac{\mu_{i0}}{\mathrm{shape}}\right)\;\\ v_{ij}=\mathrm{Possion}\left(\mu_{ij}\right) \end{array} \end{array} \end{array}$$

In the aforementioned equations, δ∼N(0,0.25). $r_{i0}$ is the expected count for gene i in a cell type and $L_{j}$ is the library size of sample j. Therefore, $\mu_{i0}$ is the expected count in the sample to be deconvoluted. We set $p_{t}$ in a range from 0.1 to 1 to control the noise level. Noise is added using Gamma and Poisson distributions [18].

### Evaluation metrics

In this study, we utilized multiple assessment metrics in a comprehensive manner to enable an objective evaluation of various deconvolution algorithms from different perspectives. The Pearson correlation coefficient (PCC) and root mean square error (RMSE) were adopted as fundamental measures to assess the linear relationship between the actual cell-type proportions and their respective predictions across different deconvolution methods. We also utilized the mean absolute percentage error (MAPE) and symmetric MAPE (sMAPE) to evaluate the degree of deviation in the prediction of each cell type.

Furthermore, inspired by previous work [56], we also integrated two gene-level evaluation metrics and a comprehensive metric into Deconer. The first gene-level metric is the structural similarity index measure (SSIM), which integrates mean value, variance, and covariance to assess the similarity between the predicted results and the ground truth. The second gene-level metric is the Jensen–Shannon divergence (JS), which employs relative information entropy to measure the disparity between two distributions. For a given gene, a higher SSIM value or a lower JS value signifies greater prediction accuracy. For the comprehensive metric, we adopted the accuracy score (AS) as the previous study with some modifications [56]. In brief, we ranked different methods based on PCC, RMSE, MAPE, and SSIM. Then, we performed a weighted summation of the rankings for these four metrics. We set the weights of these four metrics to 1 in this study. The definitions of the aforementioned metrics are provided in File S1.

## Results

### Comparison of deconvolution algorithms for gene expression data under diverse levels of noise

When processing gene expression data, it is imperative to address various forms of data noise. For instance, the same cell type under different physiological states may exhibit varied gene expression patterns, thus introducing biological noise that is difficult to avoid. In addition, technical noise also poses a substantial challenge. This kind of noise originates from discrepancies and biases in experimental operations and data pre-processing, such as library construction and sequence alignment [57]. Both biological and technical noise can profoundly impact cell-type deconvolution from gene expression data, potentially leading to skewed or inaccurate results.

We conducted an extensive evaluation of deconvolution algorithms for bulk and single-cell gene expression data. This assessment focused on the stability of deconvolution under varying degrees of noise. For the experiments, we generated 50 samples under each noise condition and computed different metrics for each cell type. This comprehensive evaluation approach allows us to understand how these deconvolution methods perform under different levels of noise, which is a critical aspect considering the inherent noise found in real data. By generating multiple samples under various noise conditions, we can gain a holistic understanding of the robustness and reliability of these deconvolution algorithms for detecting cell types from gene expression profiling data.

**Figure 2:**
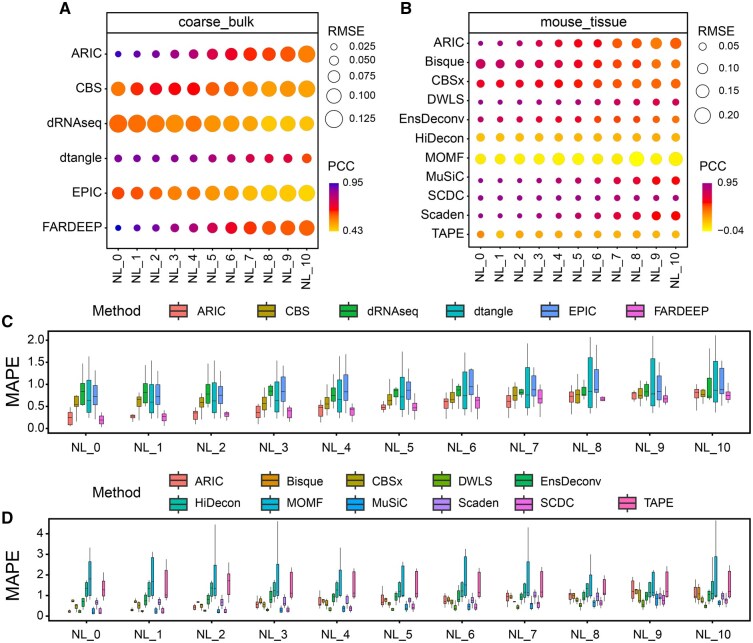
**Stability testing of various deconvolution methods in scenarios with few cell types** **A**. RMSE and PCC for methods employing bulk data as a reference. **B**. RMSE and PCC for methods employing single-cell data as a reference. **C**. MAPE for methods employing bulk data as a reference. **D**. MAPE for methods employing single-cell data as a reference. We set $p_{t}$ in a range from 0.1 to 1 to control noise levels (NL_1 to NL_10). NL_0 denotes the absence of noise incorporation. NL, noise level; NK, natural killer; CBS, CIBERSORT; dRNAseq, DeconRNASeq; CBSx, CIBERSORTx.

As shown in **Figure 2**A and B as well as Figures S2 and S3, it is evident that as data noise increases, there is a corresponding decline in performance across various deconvolution algorithms. Algorithms such as ARIC, FARDEEP, DWLS, and MuSiC demonstrate superior performance under conditions without noise. However, as noise levels intensify, these algorithms exhibit a significant increase in RMSE and a decline in PCC, consistent with our expectations. In addition, DeconRNASeq, CIBERSORT, EPIC, Bisque, and MOMF demonstrate relatively lower RMSE performance under noise-free conditions, and the PCCs of DeconRNASeq, CIBERSORT, EPIC, HiDecon, and MOMF are poorer compared to other methods. Among the selected algorithms, DWLS and Scaden exhibit remarkable robustness in scenarios with different levels of noise, maintaining consistently low RMSE levels as shown in Figure 2B. This indicates that DWLS and Scaden comprehensively address and discriminate all cellular components. In addition, MuSiC and SCDC also showcase impressive deconvolution performance, standing out among the evaluated methods.

**Figure 3:**
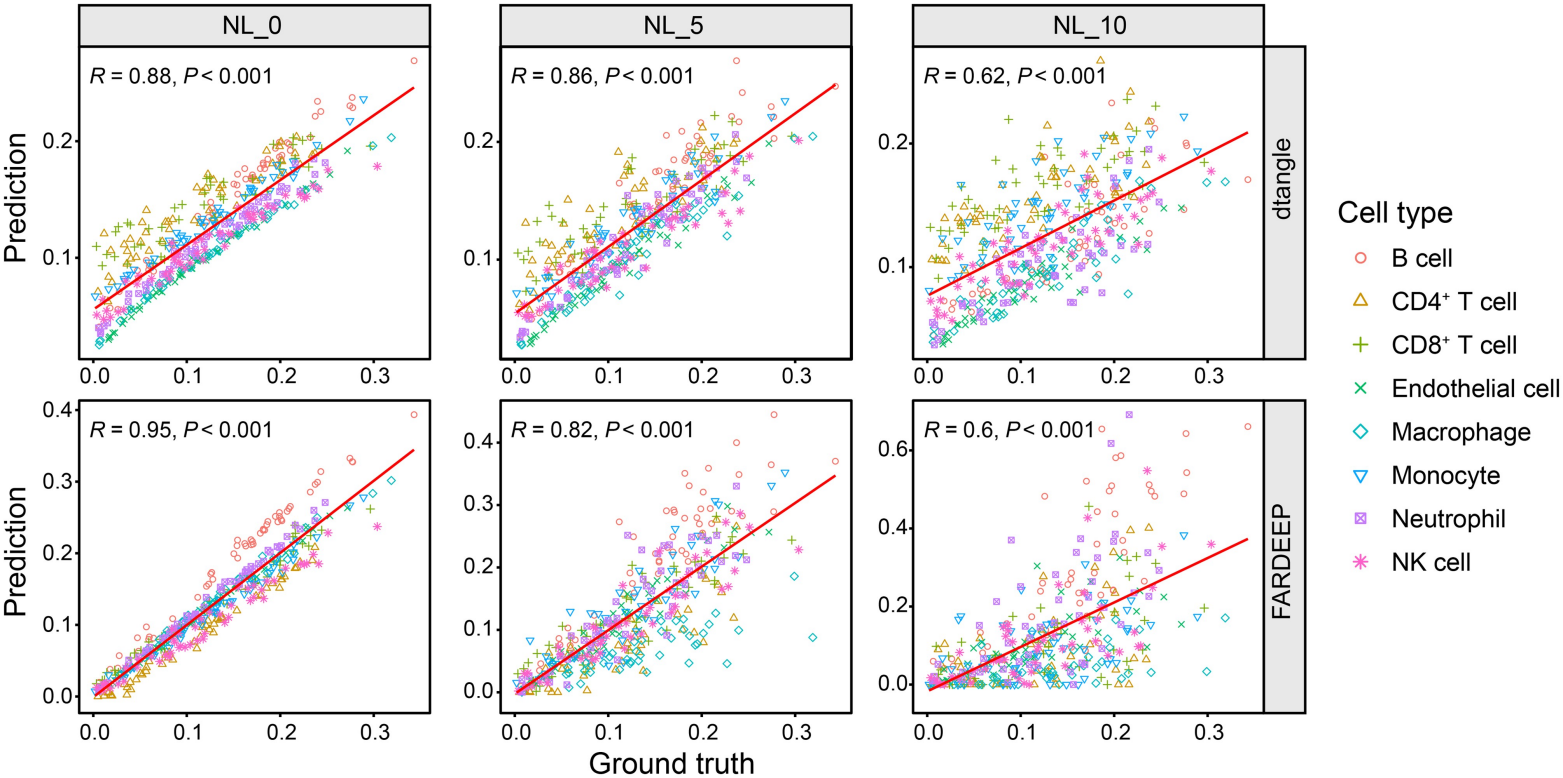
**Scatter plots for dtangle and FARDEEP**PCC and corresponding *P* value are shown in the top-left corner of each panel.

Our analysis further reveals that despite similar performance in terms of PCC and RMSE, there is a substantial discrepancy in MAPE among certain methods, as demonstrated in Figure 2C and D. This phenomenon implies the presence of cell-type biases in many methods. For instance, we generated scatter plots of dtangle and FARDEEP at three different noise levels. In Figure 2, the deconvolution results of dtangle demonstrate exceptional stability. However, its predictions exhibit a pronounced cell-type bias in **Figure 3**. Specifically, its predictions for endothelial cells and neutrophils are consistently underestimated, while CD4^+^ T cells and CD8^+^ T cells are overestimated. Similarly, the results of FARDEEP also manifest such bias, with B cells and monocytes showing noticeable deviations. Due to the unique gene expression patterns of each cell type, the limitations of deconvolution methods in capturing these intricate differences may lead to deviations between the identified cell-type proportions and their actual values. Currently, the majority of deconvolution methods resolve this issue by identifying differentially expressed genes. For instance, EPIC proposes the identification of genes specifically expressed in a single cell type [37]. However, searching for such genes is often challenging and may result in an insufficient number of markers for deconvolution. On the other hand, methods like Scaden and TAPE directly identify high-variance genes by setting a threshold, which could potentially cause an imbalance in the quantity of markers across different cell types [48,49]. Hence, the question of how to select markers to balance the accuracy of deconvolution results and the model’s attention to different cell types warrants further exploration.

### Unraveling the challenge of characterizing additional cell subtypes and building external references

In real deconvolution scenarios, the issues we encountered are substantially more complex. For example, in many research scenarios, the investigation of the proportions of subtype cells within a certain cell type is particularly significant [58–60]. Furthermore, without high-quality external references during the deconvolution process, we can only rely on limited cell expression data that often suffer from issues such as batch effects and heterogeneous data origins, posing significant challenges to the deconvolution process [12]. These issues are prevalent in almost all deconvolution studies, with batch effects and experimental origin differences being extensively discussed [12,48,60,61]. Here, we primarily delved into a detailed discussion concerning about more cell subtypes and the difficulties encountered in external reference construction.

This study utilizes four datasets (coarse_bulk, fine_bulk, mouse_tissue, and human_PBMC) for simulation and deconvolution. The coarse_bulk and mouse_tissue datasets comprise fewer cell types, and the similarity among these cell types is relatively weak. In contrast, fine_bulk and human_PBMC encompass a broader array of cell types (14 and 13, respectively), with several cell types being further divided into more subtypes. For instance, the coarse_bulk dataset contains B cells, CD4^+^ T cells, and CD8^+^ T cells, while in the fine_bulk dataset, these three cell types are further subdivided into memory B cells, naïve B cells, memory CD4^+^ T cells, naïve CD4^+^ T cells, memory CD8^+^ T cells, naïve CD8^+^ T cells, and regulatory T cells. The functionally similar cell types share similar gene expression patterns, which can lead to increased cell-to-cell confusion in the deconvolution process [60,62,63]. Unlike the mouse_tissue dataset where each cell type has 1500 high-quality sequenced cells, the human_PBMC dataset exhibits significant variation in cell numbers across cell types. This disparity renders the external reference construction less stable, thereby posing challenges to the deconvolution process. We intentionally escalated the difficulty of our comparison, utilizing the fine_bulk and human_PBMC datasets to assess the efficacy of the deconvolution methods.

Our analysis revealed notable performance disparities (Figures S4–S8; Table S3). CIBERSORT, CIBERSORTx, and SCDC maintained performance trends similar to those observed previously, demonstrating a clear decrease in deconvolution efficiency as noise levels increased. ARIC, FARDEEP, DWLS, and MuSiC continued to demonstrate exceptional performance even when confronted with an increased number of cell subtypes. The stable performance might be attributed to the specific designs of these deconvolution methods. ARIC and DWLS emphasize the consideration of rare components, while FARDEEP and MuSiC opt for the selection of specific features through outlier filtering or tree-based methods. These two aspects are particularly crucial for accurate deconvolution of cell subtypes. The results of dtangle remained stable, consistent with the findings presented in Figure 2. It is noteworthy that two deep learning-based methods, Scaden and TAPE, exhibited relatively stable performance. Both Scaden and TAPE are built upon deep neural networks, which are exemplary model-free machine learning techniques with strong adaptability to data [48,49,64]. These methodologies leverage single-cell data to generate numerous mixed samples for model training, which guarantees that they are capable of maintaining their performance, even when faced with more complex data and special conditions, highlighting their potential utility in the context of cell deconvolution from gene expression data.

However, in the human_PBMC dataset, the prediction accuracy for the proportions of certain rare cell types, such as plasmacytoid dendritic cells (pDCs) and myeloid dendritic cells (mDCs), is substantially compromised (Figure S8). These cell types are represented in very low quantities, presenting considerable challenges in effectively simulating their proportional presence under complex conditions. This scarcity of specific cell types in the dataset contributes to a decreased prediction accuracy. Therefore, it is crucial for deep learning-based methods to ensure the availability of a sufficient number of high-quality single cells for the generation of simulated samples in training. The quality of the single-cell data directly influences the learning process, where the representativeness and diversity of the data are paramount for the model to understand complex patterns and make accurate predictions. The utilization of a larger set of high-quality single cells increases the robustness of the training procedure, thereby enhancing the overall reliability and predictive power of the model. This data enrichment allows for better generalization when the model encounters complex situations, such as predicting the proportions of specific cell types in a given sample. Therefore, the usage of a sufficient number of high-quality single cells is not just beneficial but essential for training a deep learning model to ensure its reliability and validity in diverse and complex scenarios.

Additionally, we found that the prediction bias was almost invariably observed in all methods (Figures S7 and S8). Among these, ARIC, FARDEEP, DWLS, and MuSiC, which inherently incorporate gene feature selection mechanisms, demonstrated superior deconvolution performance. In contrast, other methods that do not natively incorporate feature selection exhibited more conspicuously biases (Figures S7 and S8). Scaden and TAPE, which directly use highly variable genes, showed even more pronounced cell-type biases. Hence, when estimating proportions for finer cell subtypes, careful selection of marker genes is imperative; otherwise, biases stemming from cell-type similarities are extremely likely to occur.

### Deconvolution of rare components

Accurately estimating the proportions of specific cell types, especially rare cell types in certain circumstances, is of crucial importance in uncovering their significance in specific phenotypes or diseases [4,20]. For instance, precise estimation of the proportion of tumor-infiltrating lymphocytes (TILs) plays a vital role in predicting clinical prognosis and developing personalized treatment strategies [65–67]. TILs are lymphocytes present in tumor tissues and are critical components of the immune system. They interact with tumor cells, regulating and influencing tumor growth, progression, and treatment response. TILs encompass various immune cell subtypes, including CD8^+^ T cells, CD4^+^ T cells, and natural killer (NK) cells [68,69]. However, studies have revealed variations in the proportions of TILs among patients with the same cancer type. A tumor tissue with the presence of TILs and other immune cells is referred to as a hot tumor. Hot tumors with a high proportion of TILs are known to exhibit strong immune responses, which are often associated with favorable prognosis and increased sensitivity to immunotherapy. In contrast, cold tumors lack significant TILs and other immune cells in the tumor tissue, resulting in a relatively weak immune response. Cold tumors are typically associated with poor prognosis and low sensitivity to immunotherapy. Therefore, accurate analysis of the proportions of TILs in the tumor microenvironment of different patients is crucial for understanding the immune characteristics of tumors and providing important guidance for clinical classification and treatment strategies. In addition, research on circulating cell-free RNA (cfRNA) also requires the accurate estimation of the proportion of rare cell types [70–73]. cfRNA originates from processes such as cell apoptosis and secretion [71,73]. Due to their active division and metabolism, cancer cells release cfRNA into the blood. Certain genes known as “dark channel biomarkers” have been found to retain tissue-specific and cancer-specific characteristics in cfRNA [74]. This discovery provides new avenues for future cancer detection, prediction of tumor tissue origin, and determination of cancer subtypes. However, in early-stage cancer patients, the size of the lesion tissue is small, resulting in an extremely low proportion of cfRNA released by cancer cells into the blood, making detection extremely challenging.

For this problem, we conducted tests on the presence of rare components using the aforementioned deconvolution methods and evaluated the sensitivity of different algorithms to rare components. Following the approach used by Song et al. [12], we set up a gradient of proportions to simulate different scenarios of rare components. The gradient proportions were set as 0.001, 0.003, 0.005, 0.008, 0.01, 0.03, and 0.05, respectively. Due to the small scale of the simulation proportions, MAPE can yield large percentage errors for predicted values that are zero or close to zero, potentially causing biased evaluation results. Therefore, we chose to use sMAPE to measure the relative prediction error.

**Figure 4:**
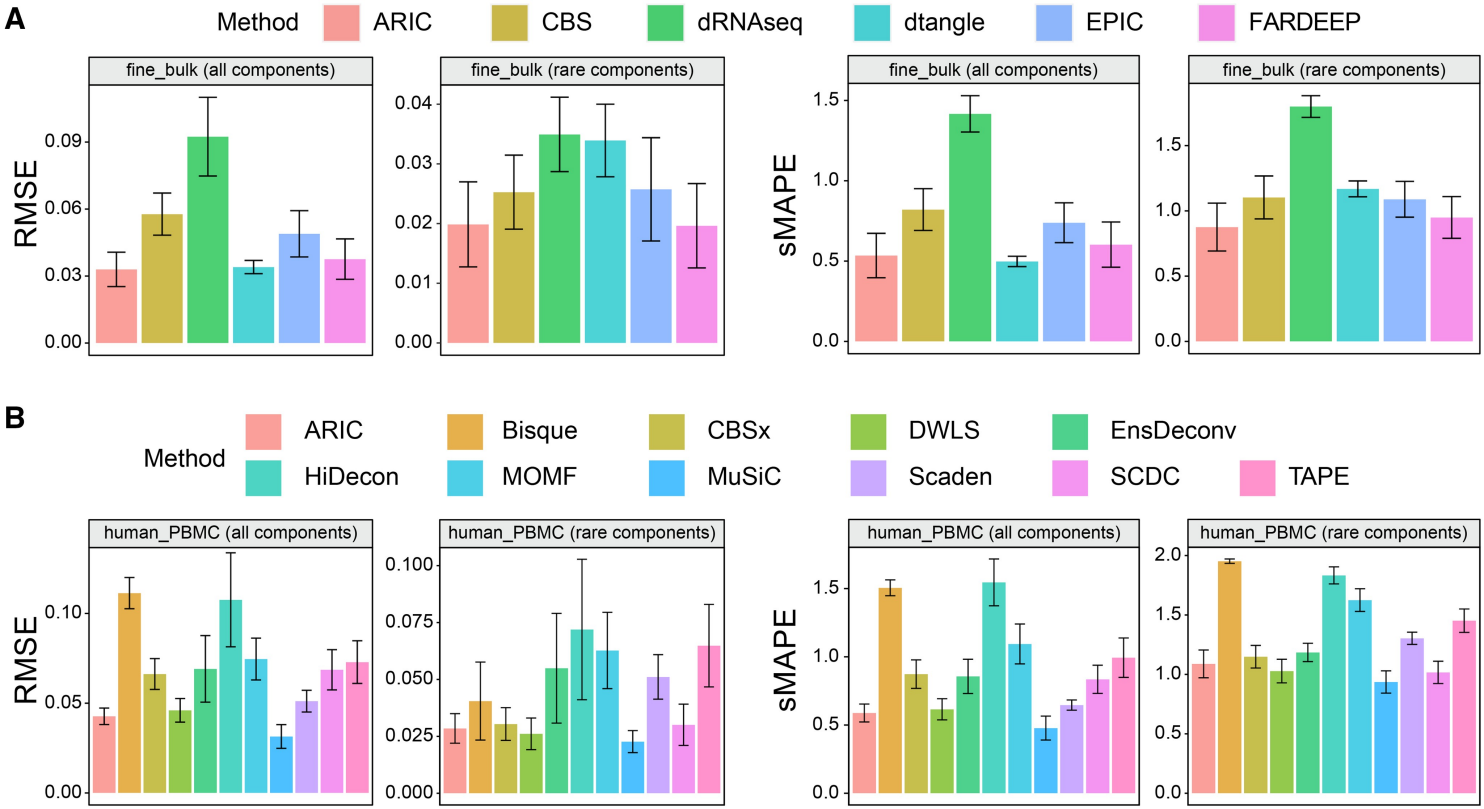
**Impact of rare components on deconvolution performance using fine_bulk and human_PBMC datasets** **A**. The results for methods employing bulk data as a reference. **B**. The results for methods employing single-cell data as a reference. All components as well as rare components are illustrated, respectively.

In our evaluation experiments, we tested the aforementioned methods on the four datasets provided by Deconer and compared their performance in terms of RMSE and sMAPE metrics. Overall, ARIC, FARDEEP, DWLS, and MuSiC demonstrate remarkable performance in predicting minor components (**Figure 4**, Figure S9). However, the results reveal that many methods tend to predict rare components as zero, like Bisque, EPIC, Scaden, and TAPE. For example, in the mouse_tissue dataset, Bisque predicted all rare components as zero (Figure S9). We hypothesize that this is because many methods focus on measuring errors such as mean absolute error (MAE), RMSE, or weighted RMSE, which tend to emphasize errors in high-proportion components while neglecting predictions for rare components. Although these methods often achieve good RMSE scores, their performance is inadequate in handling rare components, as indicated by the sMAPE metric.

Scaden and TAPE are two recently proposed deep learning-based methods that utilize random proportions for training data generation in the absence of prior knowledge [48,49]. However, these approaches face difficulties in comprehensively simulating crucial rare components. Therefore, it is preferable to perform simulation data generation based on prior information. Notably, TAPE employs the Dirichlet distribution as a tool for generating the mixture proportions in simulated data [49]. This distribution allows for flexible control of component ratios by adjusting the parameters set for the Dirichlet distribution. Users can simulate components by fine-tuning these parameters, generating data that closely align with real-world scenarios.

### Impact of training data generation on deep learning-based deconvolution methods

In recent years, deep learning-based deconvolution methods, especially Scaden, have received significant attention in numerous cutting-edge studies. Multiple studies have demonstrated their excellent performance in cell-type deconvolution across various conditions [75–77]. By harnessing the powerful feature selection capability underlying deep learning, Scaden enables automatic selection of signature genes and deconvolution of expression values by simply inputting highly variable genes. Furthermore, deep learning-based methods exhibit strong robustness to input data noise and bias, surpassing the capabilities of conventional regression-based deconvolution methods [48]. Building upon Scaden, TAPE has made further advancements by employing an autoencoder to reconstruct gene expression profiles, thereby enhancing the robustness [49]. Although these deep learning-based methods have achieved remarkable success, our analysis reveals the significant impact of data generation approaches on the effectiveness of these methods.

For Scaden and TAPE, there are certain differences in the way they generate training data. Specifically, Scaden directly samples and normalizes from a uniform distribution, which is similar to the approach used in some other studies [4,15]. On the other hand, TAPE generates random proportions using the Dirichlet distribution, which can better simulate real-world scenarios when there are certain prior proportions [49]. However, in most cases, obtaining accurate prior proportions is challenging. Therefore, in general, these two data generation approaches struggle to achieve good coverage in the proportion space, especially when there are a large number of cell types, making it difficult to accurately detect cell types under extreme proportion conditions. Additionally, since the generation of training data relies on external single-cell sequencing datasets, it is important to have an adequate number of cells for each cell type and preferably maintain a certain level of consistency. However, this requirement contradicts the issue of cellular heterogeneity in single-cell data [78]. Thus, the careful selection of single-cell data for training becomes crucial.

**Figure 5:**
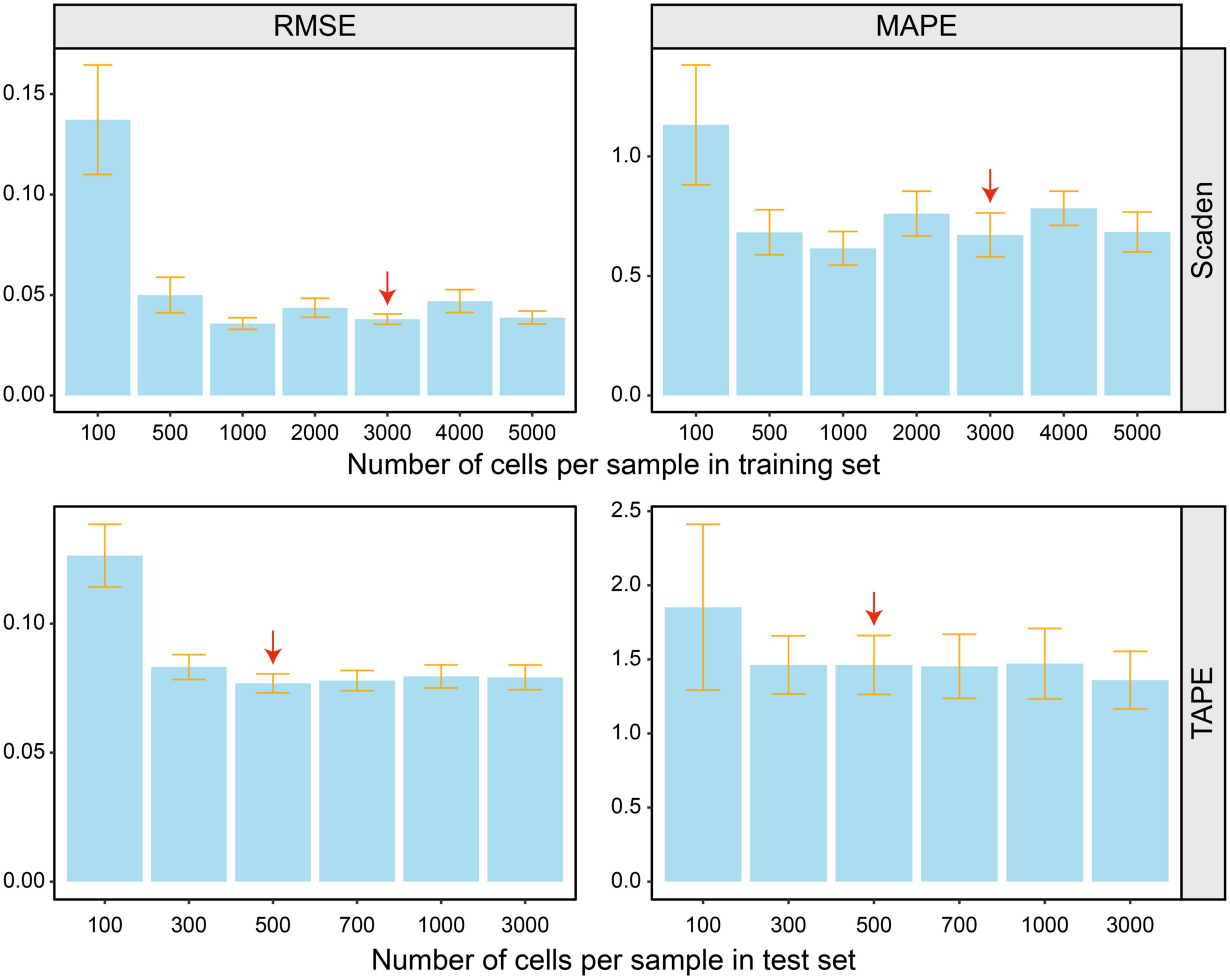
**Impact of simulated cell numbers on deconvolution performance**The top panel shows the results for Scaden and the bottom panel shows the results for TAPE. The red arrow means that the cell numbers used for generating training and test samples are consistent.

Additionally, we found that the number of cells in mixed samples during training also has an impact on the deconvolution performance. In the Scaden method, this value is set to 100, and users can adjust it according to their needs. In contrast, the TAPE method sets this value to 500 and does not support adjustment (in the latest version 1.1.2). To verify this phenomenon, we conducted tests using the mouse_tissue dataset by using the original implementation of the two methods. For Scaden, we generated 50 pseudo-bulk samples, each consisting of 3000 single cells. We then adjusted the number of cells in the training samples generated by Scaden, setting them to 100, 500, 1000, 2000, 3000, 4000, and 5000, respectively. As for TAPE, we generated pseudo-bulk samples with cell numbers of 100, 300, 500, 700, 1000, and 3000, and kept the mixed cell quantity during training consistently at 500. Through this experimental design, we can observe the effect of the number of cells in mixed samples during training on the deconvolution performance. This will help us understand the performance of Scaden and TAPE under different cell mixing scenarios and their applicability with various parameter settings. As shown in **Figure 5**, when the cell number for generating the training samples is less than the number of test samples, both Scaden and TAPE exhibit a noticeable decrease in deconvolution accuracy and stability. This effect is particularly evident when the cell number in the training samples is set to 100. However, when the number of cells in the training samples exceeds 1000, the overall performance of Scaden and TAPE tends to stabilize. This phenomenon is likely attributed to the heterogeneity of single-cell data. When the number of cells used to generate the training data is small, the model struggles to learn a stable gene expression profile for deconvolution, resulting in a decrease in effectiveness. In general, bulk data generated in scientific or clinical fields often comprise a vast number of cells, typically far exceeding the order of thousands or tens of thousands. Therefore, we recommend collecting a large amount of high-quality single-cell data and increasing the number of cells when generating training samples when utilizing deep learning methods for deconvolution.

### Potential applications and challenges of cell-type deconvolution methods for gene expression data

Cell-type deconvolution holds significant promise for applications in clinical medicine. By deciphering the changes in cell-type proportions, we can gain a deeper understanding of disease progression and pathophysiological mechanisms, providing valuable insights for disease diagnosis, treatment, and prognosis [28,43,60].

Cell-type deconvolution can aid in identifying specific biomarkers for particular diseases, enabling early diagnosis. By analyzing the changes in the proportions of different cell types, disease-related features can be distinguished [42,79]. For instance, there is an increased proportion of tumor cells in cancer or alterations in the proportions of specific immune cell subsets associated with immune-related disorders [37,80]. In rheumatoid arthritis, variations in the proportions of synovial fibroblasts and immune cell subtypes such as T cells, B cells, and NK cells exist among fibroid, lymphoid, and myeloid types [81]. This provides a crucial foundation for precision medicine, facilitating the development of individualized clinical treatments and interventions.

Cell-type deconvolution can also reveal features and patterns of disease progression [20,79]. In many diseases, such as chronic conditions and autoimmune disorders, the changes in the proportions of specific cell types are closely associated with disease progression and severity [4,81–83]. By deciphering the proportion changes of different cell types, we can observe the enrichment or depletion of disease-relevant cell subpopulations, providing insights into disease stages, pathological characteristics, and potential molecular mechanisms. This monitoring can assist in evaluating treatment efficacy, making timely adjustments to treatment plans, maximizing patient survival rates, and improving life quality. For example, alterations in the proportions of proximal tubule (PT) cells and renal interstitial fibroblasts can reflect the progression of chronic kidney disease [82,83]. Changes in TIL proportions can reflect the clinical efficacy of cancer immunotherapy [84–86]. Furthermore, cell-type deconvolution provides an important tool for gaining in-depth insights into the pathogenesis of diseases and evaluating drug efficacy during the drug development process [87]. By comparing the changes in cell composition across different disease states, we can uncover the underlying mechanisms, key signaling pathways, and regulatory networks involved in the disease. This aids in identifying novel therapeutic targets and developing innovative treatment strategies, driving advancements in disease research.

**Figure 6:**
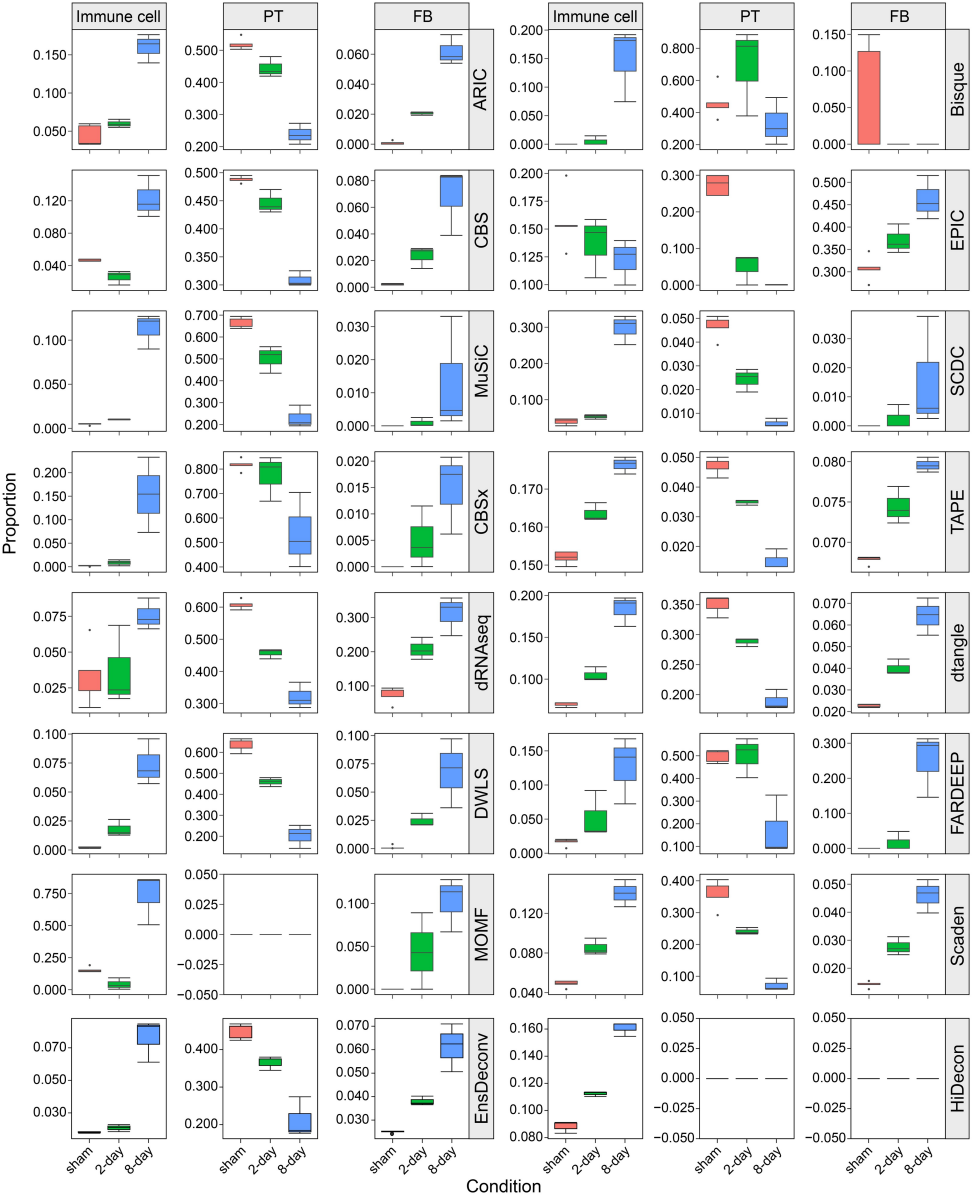
**Deconvolution results of each method in the UUO mouse model**UUO, unilateral ureteric obstruction; PT, proximal tubule cell; FB, fibroblast.

Despite the considerable attention and development of deconvolution models, inconsistencies in the deconvolution results still exist among different methods in practical applications. To address this issue, we employed the unilateral ureteric obstruction (UUO) mouse model [34], as used in a previous study [4], to evaluate the 16 deconvolution models mentioned above. The UUO mouse model serves as an experimental simulation of chronic kidney disease (CKD) and consists of three groups of bulk RNA-seq data from renal tissues collected at different time points: sham-operated, 2-day post-ligation, and 8-day post-ligation [34]. In the experiments, various phenomenon-related changes in cell-type proportions are already known [4,47,88–90]. For example, low-grade inflammation reflects an increase in immune cell proportions, while the development of glomerulosclerosis and renal interstitial fibrosis is associated with a decline in PT cell proportion and an increase in fibroblast proportion. As our former work [4], we predicted the proportions of immune cells (summation of macrophages, T lymphocytes, B lymphocytes, and NK cells), PT cells, and fibroblasts at different stages for each method. As shown in **Figure 6**, most methods are capable of predicting the trends in cell-type proportion changes mentioned above. However, there are significant variations in the identified cell-type proportions. For instance, methods such as Scaden, TAPE, FARDEEP, CIBERSORTx, and ARIC accurately capture the changing trends in cell-type proportions, while CIBERSORT, MOMF, and EPIC exhibit some prediction biases (Figure 6, Figure S10). Nevertheless, several methods demonstrate consistent predictions, including ARIC, FARDEEP, and Scaden (Figure 6). Considering the lack of prior knowledge about specific cell-type proportions in most applications and individual differences, it is crucial to employ different deconvolution methods for the same sample. Obtaining consistent results across multiple deconvolution methods will enhance the reliability of the analysis.

## Discussion

**Figure 7:**
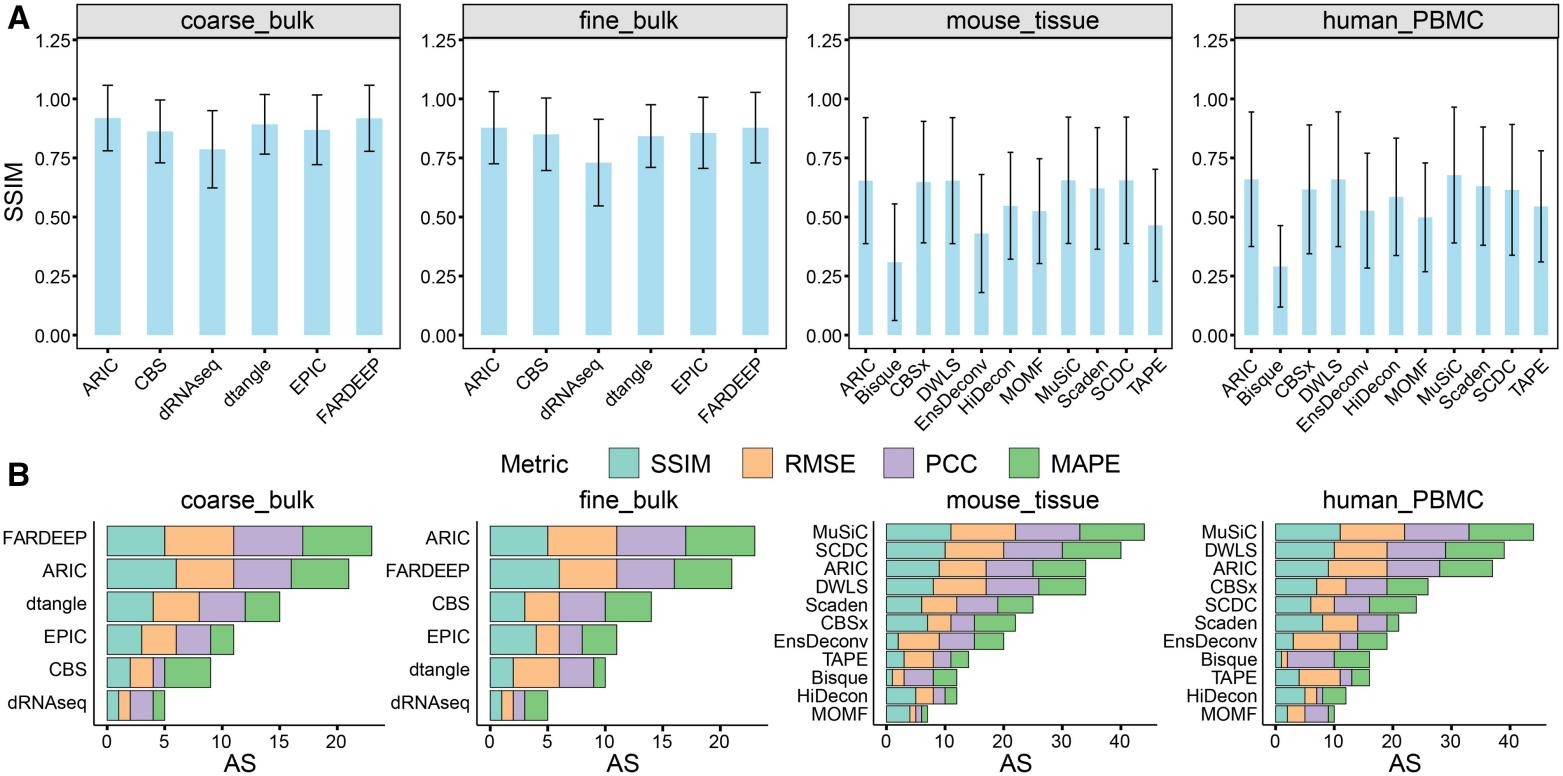
**The results of SSIM and AS for different datasets** **A**. The gene-level metric for different methods. The SSIM value is computed without artificial noise. The error bar represents the standard deviation. **B**. The AS value for different methods. The AS values integrate multiple metrics for different methods, and are ranked in descending order across methods. AS, accuracy score.

The analysis under different conditions highlights the characteristics and stability of benchmarked algorithms. Naturally, our analysis of cell-type deconvolution is not limited to this. In addition to using PCC, RMSE, and MAPE as metrics, Deconer also provides gene-level metrics such as SSIM and JS, as well as a comprehensive metric AS. We conducted relevant analyses on simulated data without specific artificial noise (**Figure 7**A). The results at the gene level revealed interesting phenomena. For the case where bulk data are used as a reference, since it represents an averaged gene expression signal, the SSIM values of different methods are relatively high (most methods have an average SSIM greater than 0.75). However, for the scenario where single-cell data are used as a reference, different methods struggle to achieve high SSIM values (the average SSIM for different methods is less than 0.75). We believe this situation is mainly due to the heterogeneity of single-cell data. This also indirectly indicates that the quality screening of single-cell data is crucial for methods using single-cell data as a reference. We further used the comprehensive metric AS for evaluation (Figure 7B). The results showed that methods with built-in feature selection functions, such as MuSiC, FARDEEP, DWLS, and ARIC, tend to perform better.

It is worth mentioning that cell-type deconvolution methods for gene expression data based on deep learning techniques have gained increasing popularity. Leveraging the powerful feature selection and fitting abilities of deep learning models, methods like Scaden and TAPE exhibit strong capabilities in addressing batch effect issues [48,49]. However, ensuring high-quality and diverse training data, incorporating prior knowledge of cell-type proportions, and optimizing the model with tailored weights for different cell types are critical steps to enhance the stability, accuracy, and effectiveness of these methods. Firstly, it is preferable to have high-quality external single-cell references that ensure an adequate number of cells for each cell type. If the training data used contain high variance and low cell numbers for certain cell types, it can lead to unstable results. Therefore, the quality and quantity of single-cell reference data are crucial for obtaining a stable and accurate deconvolution model. Secondly, when generating training data, it is beneficial to have some prior knowledge of the cell-type proportions, especially when dealing with a large number of cell types that need to be identified. Fully relying on random simulations may not guarantee that the model learns the correct parameter space, particularly for rare components. Lastly, in terms of model improvement, the adoption of different weights for different cell types, as demonstrated by methods like DWLS, has proven to be effective. If weights for the loss function can be adjusted specifically for different cell types within deep learning methods, it will further enhance the effectiveness of fine-tuning the deep learning models.

In addition, the significant discrepancies in the prediction results of different algorithms pose challenges to the practical application of deconvolution algorithms. In real-world applications, the proportions of each cell type are typically unknown, and large variations in the results obtained from different algorithms can reduce the credibility of deconvolution algorithms. In the analysis of cell-type deconvolution, the differences among different methods may arise from variations in algorithm principles, model assumptions, and data processing. These factors can lead to certain biases in the estimation of proportions for specific cell types. Therefore, it is necessary to consider the results of cell-type deconvolution in a comprehensive manner, such as through the adoption of ensemble learning with multiple models, and combining with prior knowledge and experimental validation for interpretation and inference. For specific diseases or research proposals, it is advisable to select multiple deconvolution methods for comparison and validation. Although there may be differences among deconvolution methods in predicting the trends of cell-type proportions, obtaining consistent results through the use of multiple methods will enhance the confidence in cell-type proportion changes and provide more reliable and comprehensive analytical outcomes.

According to the evaluation analysis in this study and considering previous relevant research [18,23,24], we provide the following recommendations for reference-based gene expression deconvolution analysis. (1) Ensure that the input data are in the correct data space. Some methods require input in TPM format, such as EPIC and ARIC, while others accept raw counts, such as Scaden and TAPE. (2) It is preferable for the deconvolution methods to have their own independent feature selection strategies. In our analysis, we found that methods with built-in feature selection strategies, such as FARDEEP and DWLS, consistently performed well. If a method does not have built-in feature selection, we recommend applying strict differential gene selection methods such as DESeq2 or DEsingle before performing deconvolution [91,92]. This not only avoids instability in deconvolution caused by issues such as collinearity but also reduces computational complexity. (3) Whenever possible, use high-quality single-cell data as an external reference for deconvolution. High-quality single-cell data play a crucial role in studying specific cell subpopulations and obtaining more refined deconvolution results. (4) Whenever possible, use multiple deconvolution methods to increase the reliability of the results. (5) Finally, deconvolution methods should be tailored to the application context. Employing different deconvolution strategies in different scenarios is key to achieving proper and accurate results. For general deconvolution problems, we recommend using methods with built-in feature selection to improve model performance, such as FARDEEP, MuSiC, and DWLS. For cases that require precise estimation of rare components, such as estimating the concentration of immune cells within the tumor microenvironment, DWLS or ARIC is recommended.

## Conclusion

In this study, we developed a unified expression data deconvolution assessment platform called Deconer and systematically evaluated 16 reference-based gene expression deconvolution algorithms. Through analysis of both simulated and real-world datasets, it was observed that algorithms such as MuSiC, FARDEEP, DWLS, and ARIC, which incorporate feature selection functions, demonstrated relatively higher accuracy and sensitivity, especially for rare components. Moreover, these methods exhibited consistency in the deconvolution results of real gene expression data. On the other hand, recent advancements in deep learning techniques, such as Scaden and TAPE, have shown enhanced stability and remarkable performance.

With the increasing availability of scRNA-seq datasets and the advancements in deep learning, cell-type deconvolution will become more precise. There is a need for more attention on specific cell types and the development of cell-type deconvolution algorithms that integrate phenotypic information. The evaluation results presented in this study will enhance the development of deconvolution algorithms, and cell-type deconvolution will be widely applied in personalized medicine and precision healthcare in the near future.

## Code availability

Deconer has been implemented as an open-source R package available at the GitHub repository (https://honchkrow.github.io/Deconer/). It has also been submitted to BioCode at the National Genomics Data Center (NGDC), China National Center for Bioinformation (CNCB) (BioCode: BT007577), which is publicly accessible at https://ngdc.cncb.ac.cn/biocode/tool/7577.

## Supplementary Material

qzaf009_Supplementary_Data

## Data Availability

The processed datasets of this study are provided at https://honchkrow.github.io/Deconer_dataset/.

## CRediT author statement

**Wei Zhang:** Conceptualization, Methodology, Software, Formal analysis, Writing – original draft, Funding acquisition. **Xianglin Zhang:** Software, Formal analysis. **Qiao Liu:** Writing – review & editing. **Lei Wei:** Supervision, Writing – review & editing. **Xu Qiao:** Writing – review & editing. **Rui Gao:** Writing – review & editing. **Zhiping Liu:** Conceptualization, Writing – review & editing, Supervision, Funding acquisition. **Xiaowo Wang:** Conceptualization, Writing – review & editing, Supervision. All authors have read and approved the final manuscript.
