# Supplementary material for: Deconer: An Evaluation Toolkit for Reference-based Deconvolution Methods Using Gene Expression Data
Source: Genomics Proteomics Bioinformatics. 2025 Feb 18;23(1):qzaf009. doi: 10.1093/gpbjnl/qzaf009 (PMC12221868; doi:10.1093/gpbjnl/qzaf009)
Supplement: qzaf009_Supplementary_Data [file qzaf009_supplementary_data.zip › Figure S7.pdf]

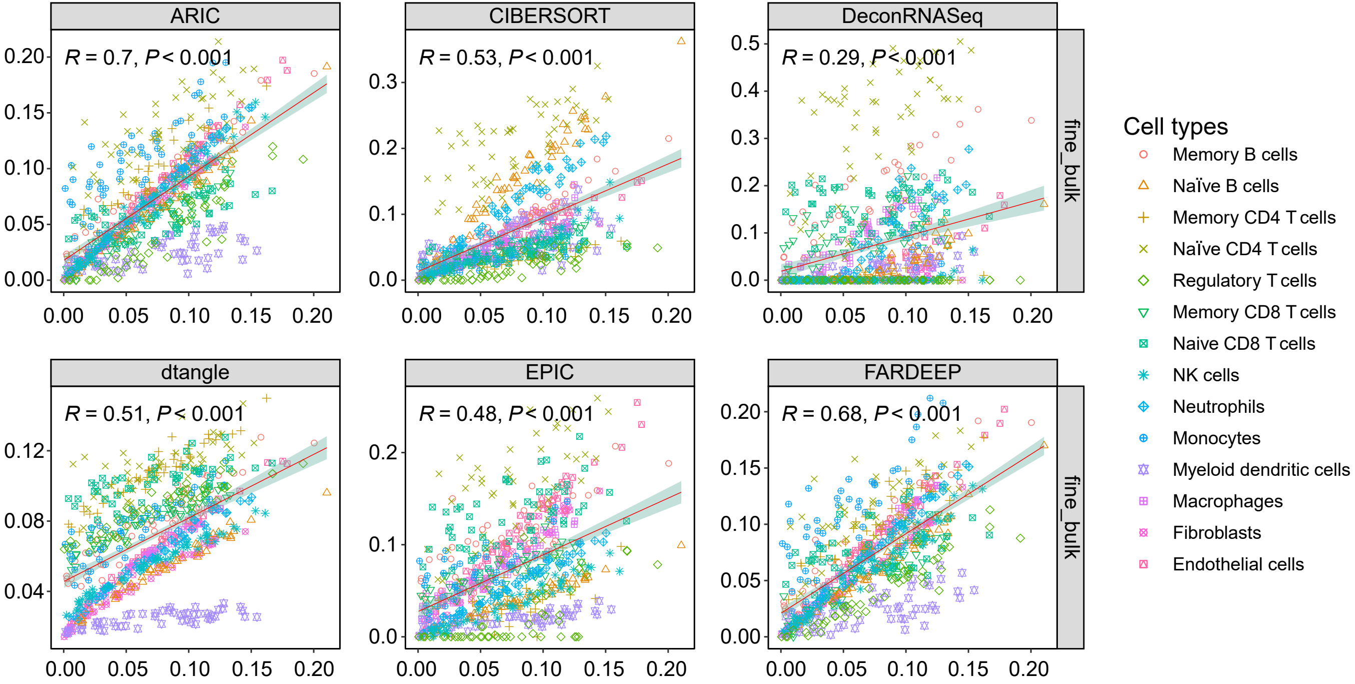
ARIC
CIBERSORT
DeconRNASeq
dtangle
EPIC
FARDEEP
R = 0.7, P < 0.001
R = 0.53, P < 0.001
R = 0.29, P < 0.001
R = 0.51, P < 0.001
R = 0.48, P < 0.001
R = 0.68, P < 0.001
fine_bulk
fine_bulk
0.00
0.05
0.10
0.15
0.20
0.0
0.1
0.2
0.3
0.4
0.5
0.04
0.08
0.12
Cell types
Memory B cells
Naïve B cells
Memory CD4 T cells
Naïve CD4 T cells
Regulatory T cells
Memory CD8 T cells
Naive CD8 T cells
NK cells
Neutrophils
Monocytes
Myeloid dendritic cells
Macrophages
Fibroblasts
Endothelial cells
