# Supplementary material for: Deconer: An Evaluation Toolkit for Reference-based Deconvolution Methods Using Gene Expression Data
Source: Genomics Proteomics Bioinformatics. 2025 Feb 18;23(1):qzaf009. doi: 10.1093/gpbjnl/qzaf009 (PMC12221868; doi:10.1093/gpbjnl/qzaf009)
Supplement: qzaf009_Supplementary_Data [file qzaf009_supplementary_data.zip › Figure S9.pdf]

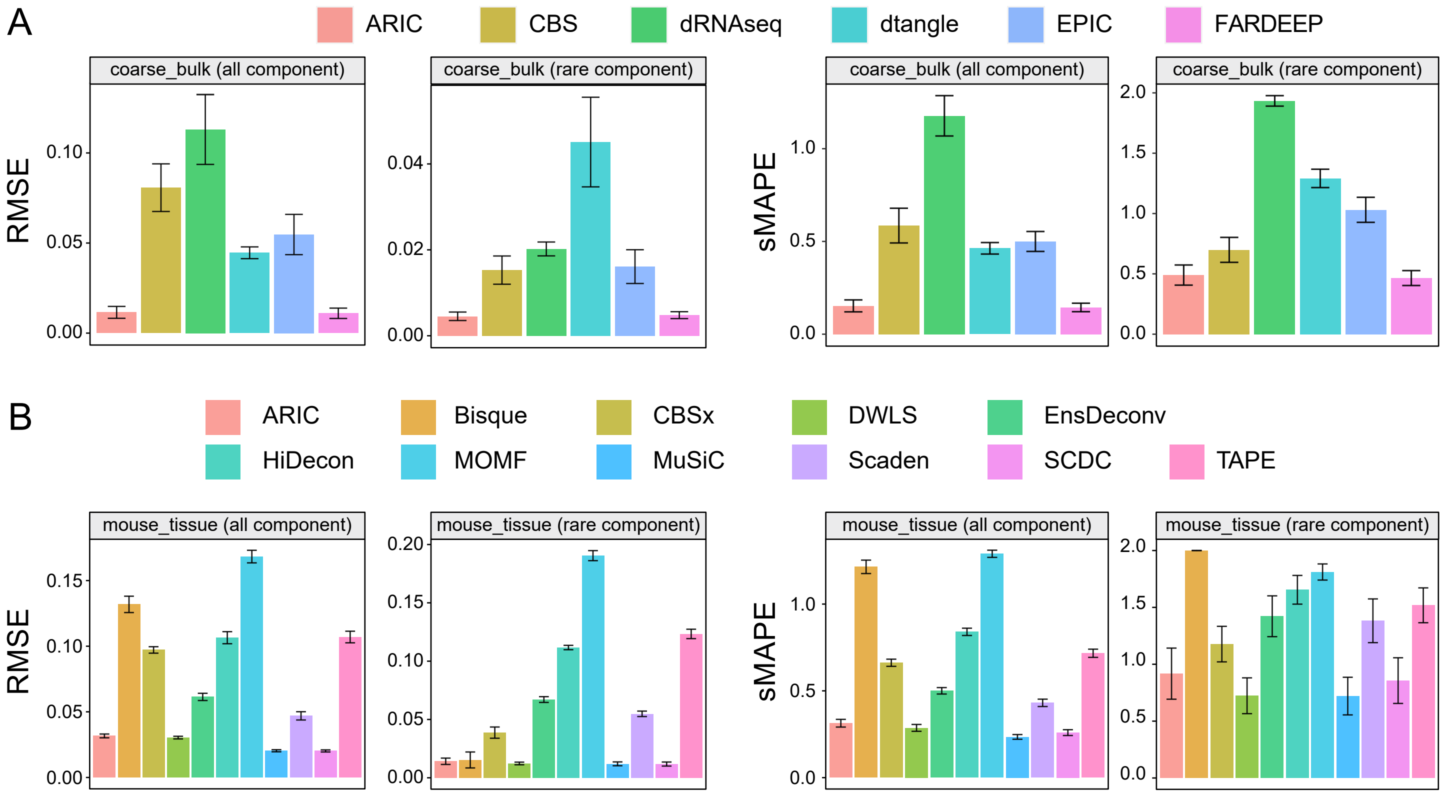
A
ARIC
CBS
dRNAseq
dtangle
EPIC
FARDEEP
coarse_bulk (all component)
coarse_bulk (rare component)
RMSE
sMAPE
0.00
0.05
0.10
0.02
0.04
0.0
0.5
1.0
1.5
2.0
B
ARIC
Bisque
CBSx
DWLS
EnsDeconv
HiDecon
MOMF
MuSiC
Scaden
SCDC
TAPE
mouse_tissue (all component)
mouse_tissue (rare component)
0.15
0.20
