# Supplementary material for: Deconer: An Evaluation Toolkit for Reference-based Deconvolution Methods Using Gene Expression Data
Source: Genomics Proteomics Bioinformatics. 2025 Feb 18;23(1):qzaf009. doi: 10.1093/gpbjnl/qzaf009 (PMC12221868; doi:10.1093/gpbjnl/qzaf009)
Supplement: qzaf009_Supplementary_Data [file qzaf009_supplementary_data.zip › File S1.docx]

# File S1 Supplementary methods and results

## Section 1 Package “Deconer”

### *Section 1.1 Introduction*

Cell type proportion is related to phenotypes or diseases [1,2]. Therefore, quantifying cell or tissue proportions is important for understanding the mechanisms in biological processes. Here, we proposed a cell type deconvolution evaluating toolkit named Deconvolution Evaluator (Deconer) to perform comprehensive and systematic evaluation of different algorithms.

Deconer consists of 6 main part functions as below.

- Pseudo bulk data generation (from massive bulk data and single-cell data).
- Stability analysis under different types of noise.
- Rare component analysis.
- Unknown component analysis.
- Comprehensive evaluation metrics as well as pretty figure generation.
- Well-characterized datasets for deconvolution utilities.

For more information, please see the following pages.

- [Deconer homepage](https://honchkrow.github.io/Deconer/)
- [Deconer manual](https://honchkrow.github.io/Deconer/inst/documents/Deconer_manual.pdf)
- [Deconer vignettes](https://honchkrow.github.io/Deconer/inst/documents/Deconer_intro.html)
- [Deconer dataset](https://honchkrow.github.io/Deconer_dataset/)

### *Section 1.2 Installation*

Deconer is based on R and can be easily installed on Windows, Linux, as well as macOS.

First, users should install R >= 4.1.0.

Next, install devtools and Deconer.

### *Section 1.3 Functions used in this study*

We generated all the simulated data using Deconer package, including pseudo bulk data generated from massive RNA sequencing (RNA-seq) data as well as from single-cell RNA-seq (scRNA-seq) data (see [Deconer homepage “Section 3: Gnerating Pseudo Bulk Data](https://honchkrow.github.io/Deconer/#section-3-gnerating-pseudo-bulk-data)”). For noise analysis, please see Deconer homepage “[Section 4: Noise Analysis](https://honchkrow.github.io/Deconer/#section-4-noise-analysis)”. For rare component analysis, please see Deconer homepage “[Section 5: Rare Component Analysis](https://honchkrow.github.io/Deconer/#section-5-rare-component-analysis)”. In addition, most of the figures are also generated directly from Deconer (see Deconer homepage “[Code Demo 1: Evaluating The Deconvolution Results In A Simple Manner](https://honchkrow.github.io/Deconer/#code-demo-1-evaluating-the-deconvolution-results-in-a-simple-manner)” and “[Code Demo 2: Evaluating The Deconvolution Results For Multiple Method](https://honchkrow.github.io/Deconer/#code-demo-2-evaluating-the-deconvolution-results-for-multiple-method)”).

### *Section 1.4 Datasets*

Besides, we collected well-characterized deconvolution datasets for users. Some of them are known cell type proportions. Some datasets without know the true proportion, but the related phenotype can be accessed. We provide the processed datasets with bulk data, reference data, as well as true proportions, which means that these datasets can be used directly. We summarized the datasets in the Table S1.

## Section 2 Criteria for selecting benchmarking methods

We conducted a survey on commonly used deconvolution methods. Below is a summary of some algorithms, along with explanations for our selection of certain models.

Firstly, we focused on deconvolution algorithms based on bulk data. Common methods include Tumor IMmune Estimation Resource (TIMER) [3], Digital Sorting Algorithm (DSA) [4], xCell [5], Microenvironment Cell Populations-counter (MCP-counter) [6], DeconRNASeq [7], Estimating the Proportion of Immune and Cancer cells (EPIC) [8], dtangle [9], Fast And Robust DEconvolution of Expression Profiles (FARDEEP) [10], and Cell-type Identification By Estimating Relative Subsets Of RNA Transcripts (CIBERSORT) [11]. These methods mainly model based on the principle of linear superposition of gene expression data. For example, CIBERSORT is a well-known deconvolution method that uses linear ν-support vector regression (ν-SVR). TIMER is based on a linear regression model and provides markers for different tumor microenvironments, making it commonly used in tumor mechanism analysis. Zhong et al. proposed the Digital Sorting Algorithm, which has a special selection for cell type-specific genes. DeconRNASeq, xCell, MCP-counter, and EPIC are all based on the least square linear regression model. However, EPIC has high requirements for feature selection and is robust to unknown mixture content. dtangle is a newly proposed method that is robust to outliers. FARDEEP adopts the least trimmed squares method and is also robust to outliers.

Single-cell-based deconvolution methods include ARIC [2], Bisque [12], CIBERSORTx [13], dampened weighted least squares (DWLS) [14], Multi-Omics Matrix Factorization (MOMF) [15], MUlti-Subject SIngle Cell deconvolution (MuSiC) [1], single cell–assisted deconvolutional deep neural network (Scaden) [16], Tissue-AdaPtive autoEncoder (TAPE) [17], Hierarchical Deconvolution (HiDecon) [18], Ensemble Deconvolution (EnsDeconv) [19], and SCDC [20]. Among these, ARIC can be used for bulk, single-cell, and DNA methylation data analysis. CIBERSORTx is based on linear ν-SVR and is an upgraded version of CIBERSORT. Bisque uses non-negative least squares (NNLS) regression. DWLS and MuSiC employ different least squares methods. MOMF is the only model that uses the Poisson distribution to describe gene expression. HiDecon guides deconvolution by constructing a differentiation tree. Scaden and TAPE are the latest methods based on deep learning. Scaden uses three different fully connected neural networks for deconvolution. TAPE incorporates gene expression data reconstruction while using neural network deconvolution. EnsDeconv and SCDC are ensemble learning methods. SCDC performs deconvolution based on different references, while EnsDeconv integrates multiple algorithms for improved performance.

We carefully considered the selection of benchmarking methods. Our goal was to choose representative mathematical models with distinct characteristics. Here are the criteria for our selection: (1) We chose methods that utilize different mathematical models to guarantee diversity. For example, we included CIBERSORT and CIBERSORTx based on ν-SVR, DWLS using dampened weighted least squares, and FARDEEP employing least trimmed squares. Additionally, we selected methods using probabilistic models such as MOMF, deep learning models like Scaden and TAPE, and ensemble learning models such as SCDC, each with unique modelling characteristics. (2) Most of these methods have been widely applied in numerous studies. For instance, DeconRNASeq, Scaden, and DWLS have each been cited hundreds of times, while CIBERSORT and CIBERSORTx have been cited thousands of times. Therefore, these algorithms are representative. (3) Some methods were introduced in recent years and have not yet been extensively compared. Examples include Scaden (2020, the first deconvolution method using deep learning), ARIC (published in 2021, applicable to bulk, scRNA-seq, as well as DNA methylation data), and TAPE (published in 2022, employing an autoencoder structure for deconvolution).

## Section 3 Datasets and pre-processing

## *Section 3.1 Bulk RNA-seq*

We collected 233 high-quality bulk RNA-seq samples to simulate different stromal populations, including immune populations (Table S2). Drawing inspiration from the [Tumor Deconvolution DREAM Challenge organized by Synapse](https://www.synapse.org/#!Synapse:syn15589870/wiki/592761), we created two distinct scenarios referred to as “coarse” and “fine”. In the coarse scenario, the mixed samples comprised 8 different cell types: B cells, CD4 T cells, CD8 T cells, endothelial cells, macrophages, monocytes, neutrophils, and natural killer (NK) cells. In the fine scenario, the mixed samples encompassed 14 different cell types: memory B cells, naive B cells, memory CD4 T cells, active CD4 T cells, regulatory T cells, memory CD8 T cells, naive CD8 T cells, NK cells, neutrophils, monocytes, myeloid dendritic cells, macrophages, fibroblasts, and endothelial cells. During data collection, we made efforts to ensure that each cell type originated from at least two different studies and that all samples were paired-end data. This approach aimed to mitigate batch effects arising from variations between single-end and paired-end sequencing experiments. It is evident that in the “fine” dataset, various cell types are further subdivided into subtypes, which often share similar biological functions. Subtypes with similar functions generally exhibit comparable gene expression profiles, making it more challenging to accurately estimate the proportions of these subtype cells.

For data pre-processing, we employed the following RNA-seq data processing workflow. In simple terms, we downloaded the FASTQ files for all samples and used AdapterRemoval to remove adapter sequences [21]. Then, we utilized HISAT2 to align all transcriptome samples to the hg19 reference genome with the parameters “-q --no-mixed --no-discordant”, and obtained BAM files using SAMtools [22,23]. During this process, Quality Control for RNA-Seq (QuaCRS) and iSeqQC were employed for quality control, and low-quality and low-depth data were directly discarded [24,25]. Subsequently, we converted the BAM files into gene expression counts using featureCounts [26].

## *Section 3.2 scRNA-seq*

We collected 10K single-cell data of human PBMCs from 10X Genomics. For this dataset, we used multimodal omics analysis (MUON)to annotate 13 reliable cell types [27]. Additionally, we curated a unique single-cell dataset for the generation of mixed samples. This dataset consisted of seven different tissues from fetal mouse [28]. Given the significant expression profile variations across these seven tissues, the samples generated from this dataset served as a fair benchmark for evaluating the capabilities of different algorithms. In addition to the simulated data, we also employed a well-characterized mouse kidney dataset to test all the deconvolution methods discussed in this paper. This dataset represents real data with unknown proportions, and we assessed the deconvolution methods by comparing the inferred cell proportions with known biological phenotypes or processes.

## *Section 3.3 Data transformation and normalization*

During the analysis process, all data were transformed into the required input format for each respective method. For instance, methods like EPIC and ARIC require transcripts per million (TPM) as input, while dtangle requires log-transformed data [2,8,9]. DeconRNASeq, on the other hand, requires data to be maintained in linear space to preserve the assumptions of the linear regression model [7]. Regarding data normalization, Scaden and TAPE, being deep learning methods, handle the MinMax scaling internally [16,17]. Previous studies have indicated that the zero mean and unit variance data standardization used by CIBERSORT may introduce estimation bias [10]. Therefore, we did not include data normalization beyond the required transformations for each algorithm.

## *Section 3.4 Settings for deconvolution methods*

For HiDecon, we adopted the following evolutionary tree.

1. Hematopoietic stem cell (HSC)
2. -Common myeloid progenitor (CMP)
3. -Monocyte lineage
4. -CD14+ monocytes
5. -Intermediate monocytes
6. -CD16+ monocytes
7. -Dendritic cells (DC) lineage
8. -Myeloid DC (mDC)
9. -Plasmacytoid DC (pDC)
10. -Common lymphoid progenitor (CLP)
11. -T cell lineage
12. -CD4+ naïve T cells
13. -CD4+ memory T cells
14. -CD8+ naïve T cells
15. -CD8+ activated T cells
16. -Mucosal-associated invariant T (MAIT) cells
17. -B cell lineage
18. -Naïve B cells
19. -Memory B cells
20. -NK cell lineage
21. -NK cells

# Section 4 Evaluation metrics

The definition of Pearson correlation coefficient (PCC), root mean square error (RMSE), mean absolute percentage error (MAPE), as well as symmetric MAPE (sMAPE) are as follows:

$$PCC\left( \boldsymbol{p},\boldsymbol{p}^{'} \right)=\frac{\sum_{i\in\Omega} \left( p_{i}-\bar{p} \right)\left( p_{i}^{'}-\bar{p^{'}} \right)}{\sqrt{\sum_{i\in\Omega} \left( p_{i}-\bar{p} \right)^{2}}\sqrt{\sum_{i\in\Omega} \left( p_{i}^{'}-\bar{p^{'}} \right)^{2}}} ,$$

$$RMSE\left( \boldsymbol{p},\boldsymbol{p}^{'} \right)=\sqrt{\frac{1}{\left| \Omega\right|}\sum_{i\in\Omega} \left( p_{i}-p_{i}^{'} \right)^{2}} ,$$

$$MAPE\left( \boldsymbol{p},\boldsymbol{p}^{'} \right)=\frac{1}{\left| \Omega\right|}\sum_{i\in\Omega} \left| \frac{p_{i}-p_{i}^{'}}{p_{i}} \right| ,$$

$$sMAPE\left( \boldsymbol{p},\boldsymbol{p}^{'} \right)=\frac{2}{\left| \Omega\right|}\sum_{i\in\Omega} \left| \frac{p_{i}-p_{i}^{'}}{p_{i}+p_{i}^{'}} \right| ,$$

where $p_{i}$ and $p_{i}^{'}$ denote the ground truth and the predicted proportion value, respectively. It is noteworthy that the aforementioned metrics in Deconer can be applied to various evaluation scenarios, including the assessment of all cell types in individual sample or across multiple samples, as well as individual evaluations of different cell types across multiple samples. Accordingly, the term $\Omega$ is used to denote the cell type set in each condition and $\left| \Omega\right|$ is the number of cell types. It is important to note that the sMAPE function is derived from the R package Metrics. It reaches a maximum value of 2 in cases where either the actual or predicted values are zero, or when the actual and predicted values have opposite signs.

The structural similarity index measure (SSIM) is defined by the following formula:

$$SSIM=\frac{(2\tilde{u}_{i}u_{i}+C_{1}^{2})(2cov(\boldsymbol{x}_{i}^{'},{\tilde{\boldsymbol{x}}}_{i}^{'})+C_{2}^{2})}{(\tilde{u}_{i}^{2}+u_{i}^{2}+C_{1}^{2})(\tilde{\sigma}_{i}^{2}+\sigma_{i}^{2}+C_{2}^{2})}.$$

Where $x_{i}^{'}$ is the scaled expression of gene $i$ in sample $j$. $\tilde{u}_{i}$ and $u_{i}$ are the means of the predicted and ground truth expression vectors, respectively. $\tilde{\sigma}_{i}$ and $\sigma_{i}$ are the variances of the predicted and ground truth expression vectors. $cov(\boldsymbol{x}_{i}^{'},{\tilde{\boldsymbol{x}}}_{i}^{'})$ is the covariance between the predicted and ground truth expression vectors. $C_{1}$ and $C_{2}$ are constants to stabilize the division with small denominators (0.01 and 0.03, respectively).

Jensen–Shannon divergence (JS) uses relative information entropy (Kullback–Leibler divergence) to measure the difference between two distributions. First, the spatial distribution probability $P_{i,j}$ is computed for each gene.

$$P_{i,j}=\frac{x_{ij}}{\sum_{j=1}^{M} x_{ij}}$$

where $x_{ij}$ denotes the expression of gene $i$ in sample $j$, $M$ is the total number of samples. Then, we calculate the JS value for each gene using the following equations:

$$JS=\frac{1}{2}KL\left( {\tilde{\boldsymbol{P}}}_{i} | \frac{{\tilde{\boldsymbol{P}}}_{i}+\boldsymbol{P}_{i}}{2} \right)+\frac{1}{2}KL\left( \boldsymbol{P}_{i} | \frac{{\tilde{\boldsymbol{P}}}_{i}+\boldsymbol{P}_{i}}{2} \right)$$

$$KL(a_{i}||b_{i})=\sum_{j=0}^{M} (a_{ij}\times log\frac{a_{ij}}{b_{ij}})$$

where $\boldsymbol{P}_{i}$ and ${\tilde{\boldsymbol{P}}}_{i}$ are the spatial distribution probability vectors of gene $i$ for the ground truth and the predicted result, respectively. $KL(a_{i}||b_{i})$ is the Kullback–Leibler divergence between two probability distributions $a_{i}$ and $b_{i}$, with $a_{i}$ and $b_{i}$ representing the predicted and real probabilities of gene $i$ in sample $j$, respectively. For one gene, a lower JS value indicates better prediction accuracy.

It is important to note that SSIM and JS require a sufficient sample size for accurate evaluation. However, the number of real datasets with known proportions is quite limited. This limitation significantly impacts the reliability of these metrics. Additionally, considering that both metrics demonstrate the accuracy per gene, we adopt SSIM for the assessment of different deconvolution methods in this study.

The definition of AS is

$$AS=a*{RANK}_{PCC}{+b*RANK}_{RMSE}{+c*RANK}_{MAPE}{+d*RANK}_{SSIM}$$

where $a$, $b$, $c$, and $d$ are all set to 1 in this study.

# Section 5 Signature genes selection

Multiple studies have indicated that differentially expressed genes play a crucial role in cell proportion deconvolution [2,8]. Therefore, we employed DEseq2 and DEsingle for signature gene selection [29,30]. Specifically, DEseq2 was used for differential gene screening in bulk data, while DEsingle was employed for single-cell data. In general, our selection of differentially expressed genes followed the following principles. Firstly, genes with low expression quality were excluded. Secondly, for methods like Scaden and TAPE that have their own high variance gene selection strategies, which explicitly state that separate differential gene screening is not required, we included all genes as input [16,17]. Additionally, our differential gene selection was based on the “one *vs*. others” strategy, meaning that when selecting differential genes for a particular cell type, all other cell types were treated as the control samples. For DEseq2, we utilized parameter settings with a *P* value of 0.001, fold change of 4, and baseMean greater than 10 and less than 1000. As for DEsingle, we extracted differentially expressed genes of the “DEg” type and set the *P* value threshold at 0.0001 and fold change at 4. For algorithms that do not provide their own feature selection strategies, we directly employed all identified differentially expressed genes as signature genes for deconvolution.

# Reference

[1] Wang X, Park J, Susztak K, Zhang NR, Li M. Bulk tissue cell type deconvolution with multi-subject single-cell expression reference. Nat Commun 2019;10:380.

[2] Zhang W, Xu H, Qiao R, Zhong B, Zhang X, Gu J, et al. ARIC: accurate and robust inference of cell type proportions from bulk gene expression or DNA methylation data. Brief Bioinform 2022;23:bbab362.

[3] Li T, Fan J, Wang B, Traugh N, Chen Q, Liu JS, et al. TIMER: a web server for comprehensive analysis of tumor-infiltrating immune cells. Cancer Res 2017;77:e108–10.

[4] Zhong Y, Wan YW, Pang K, Chow LM, Liu Z. Digital sorting of complex tissues for cell type-specific gene expression profiles. BMC Bioinformatics 2013;14:89.

[5] Aran D, Hu Z, Butte AJ. xCell: digitally portraying the tissue cellular heterogeneity landscape. Genome Biol 2017;18:220.

[6] Becht E, Giraldo NA, Lacroix L, Buttard B, Elarouci N, Petitprez F, et al. Estimating the population abundance of tissue-infiltrating immune and stromal cell populations using gene expression. Genome Biol 2016;17:249.

[7] Gong T, Szustakowski JD. DeconRNASeq: a statistical framework for deconvolution of heterogeneous tissue samples based on mRNA-seq data. Bioinformatics 2013;29:1083–5.

[8] Racle J, de Jonge K, Baumgaertner P, Speiser DE, Gfeller D. Simultaneous enumeration of cancer and immune cell types from bulk tumor gene expression data. Elife 2017;6:e26476.

[9] Hunt GJ, Freytag S, Bahlo M, Gagnon-Bartsch JA. dtangle: accurate and robust cell type deconvolution. Bioinformatics 2019;35:2093–9.

[10] Hao Y, Yan M, Heath BR, Lei YL, Xie Y. Fast and robust deconvolution of tumor infiltrating lymphocyte from expression profiles using least trimmed squares. PLoS Comput Biol 2019;15:e1006976.

[11] Newman AM, Liu CL, Green MR, Gentles AJ, Feng W, Xu Y, et al. Robust enumeration of cell subsets from tissue expression profiles. Nat Methods 2015;12:453–7.

[12] Jew B, Alvarez M, Rahmani E, Miao Z, Ko A, Garske KM, et al. Accurate estimation of cell composition in bulk expression through robust integration of single-cell information. Nat Commun 2020;11:1971.

[13] Newman AM, Steen CB, Liu CL, Gentles AJ, Chaudhuri AA, Scherer F, et al. Determining cell type abundance and expression from bulk tissues with digital cytometry. Nat Biotechnol 2019;37:773–82.

[14] Tsoucas D, Dong R, Chen H, Zhu Q, Guo G, Yuan GC. Accurate estimation of cell-type composition from gene expression data. Nat Commun 2019;10:2975.

[15] Sun X, Sun S, Yang S. An efficient and flexible method for deconvoluting bulk RNA-seq data with single-cell RNA-seq data. Cells 2019;8:1161.

[16] Menden K, Marouf M, Oller S, Dalmia A, Magruder DS, Kloiber K, et al. Deep learning-based cell composition analysis from tissue expression profiles. Sci Adv 2020;6:eaba2619.

[17] Chen Y, Wang Y, Chen Y, Cheng Y, Wei Y, Li Y, et al. Deep autoencoder for interpretable tissue-adaptive deconvolution and cell-type-specific gene analysis. Nat Commun 2022;13:6735.

[18] Huang P, Cai M, Lu X, Mckennan C, Wang J. Accurate estimation of rare cell-type fractions from tissue omics data via hierarchical deconvolution. Ann Appl Stat 2024;18:1178–94.

[19] Cai M, Yue M, Chen T, Liu J, Forno E, Lu X, et al. Robust and accurate estimation of cellular fraction from tissue omics data via ensemble deconvolution. Bioinformatics 2022;38:3004–10.

[20] Dong M, Thennavan A, Urrutia E, Li Y, Perou CM, Zou F, et al. SCDC: bulk gene expression deconvolution by multiple single-cell RNA sequencing references. Brief Bioinform 2021;22:416–27.

[21] Schubert M, Lindgreen S, Orlando L. AdapterRemoval v2: rapid adapter trimming, identification, and read merging. BMC Res Notes 2016;9:88.

[22] Danecek P, Bonfield JK, Liddle J, Marshall J, Ohan V, Pollard MO, et al. Twelve years of SAMtools and BCFtools. Gigascience 2021;10:giab008.

[23] Kim D, Paggi JM, Park C, Bennett C, Salzberg SL. Graph-based genome alignment and genotyping with HISAT2 and HISAT-genotype. Nat Biotechnol 2019;37:907–15.

[24] Kroll KW, Mokaram NE, Pelletier AR, Frankhouser DE, Westphal MS, Stump PA, et al. Quality control for RNA-seq (QuaCRS): an integrated quality control pipeline. Cancer Inform 2014;13:7–14.

[25] Kumar G, Ertel A, Feldman G, Kupper J, Fortina P. iSeqQC: a tool for expression-based quality control in RNA sequencing. BMC Bioinformatics 2020;21:56.

[26] Liao Y, Smyth GK, Shi W. featureCounts: an efficient general purpose program for assigning sequence reads to genomic features. Bioinformatics 2014;30:923–30.

[27] Bredikhin D, Kats I, Stegle O. MUON: multimodal omics analysis framework. Genome Biol 2022;23:42.

[28] Han X, Wang R, Zhou Y, Fei L, Sun H, Lai S, et al. Mapping the mouse cell atlas by Microwell-seq. Cell 2018;173:1091–107.e17.

[29] Love MI, Huber W, Anders S. Moderated estimation of fold change and dispersion for RNA-seq data with DESeq2. Genome Biol 2014;15:550.

[30] Miao Z, Deng K, Wang X, Zhang X. DEsingle for detecting three types of differential expression in single-cell RNA-seq data. Bioinformatics 2018;34:3223–4.
