# Supplementary material for: Deconer: An Evaluation Toolkit for Reference-based Deconvolution Methods Using Gene Expression Data
Source: Genomics Proteomics Bioinformatics. 2025 Feb 18;23(1):qzaf009. doi: 10.1093/gpbjnl/qzaf009 (PMC12221868; doi:10.1093/gpbjnl/qzaf009)
Supplement: qzaf009_Supplementary_Data [file qzaf009_supplementary_data.zip › supplementary material captions.docx]

**Supplementary materials**

**File S1 Supplementary methods and results**

**Figure S1 Cell annotation for human_PBMC dataset**

**A.** UMAP plot for cell clusters. **B.** The selected marker genes for each cell types. UMAP, Uniform Manifold Approximation and Projection; MAIT, mucosal-associated invariant T cells; NK, natural killer cells; Mono, Monocytes; mDC, myeloid dendritic cells; pDC, plasmacytoid dendritic cells.

**Figure S2 Performance metrics of different algorithms on dataset coarse_bulk**

**A.** RMSE for each method. **B.** MAPE for each method. **C.** sMAPE for each method. We set $p_{t}$ in a range from 0.1 to 1 to control noise level (NL_1 to NL_10). NL_0 denotes the absence of noise incorporation.

**Figure S3 Performance metrics of different algorithms on dataset mouse_tissue**

**A.** RMSE for each method. **B.** MAPE for each method. **C.** sMAPE for each method. We set $p_{t}$ in a range from 0.1 to 1 to control noise level (NL_1 to NL_10). NL_0 denotes the absence of noise incorporation.

**Figure S4 Stability testing of various deconvolution methods in scenarios with many cell types**

**A.** The RMSE and PCC for methods employing bulk data as a reference. **B.** The RMSE and PCC for methods employing single-cell data as a reference. We set $p_{t}$ in a range from 0.1 to 1 to control noise level (NL_1 to NL_10). NL_0 denotes the absence of noise incorporation.

**Figure S5 Performance metrics of different algorithms on dataset fine_bulk.**

**A.** RMSE for each method. **B.** MAPE for each method. **C.** sMAPE for each method. We set $p_{t}$ in a range from 0.1 to 1 to control noise level (NL_1 to NL_10). NL_0 denotes the absence of noise incorporation.

**Figure S6 Performance metrics of different algorithms on dataset human_PBMC**

**A.** RMSE for each method. **B.** MAPE for each method. **C.** sMAPE for each method. We set $p_{t}$ in a range from 0.1 to 1 to control noise level (NL_1 to NL_10). NL_0 denotes the absence of noise incorporation.

**Figure S7 Deconvolution results for fine_bulk datasets**

This figure corresponds to NL_0 in the Figure S5, which means that noise is not added in this test. The datasets are annotated on the right side of the graph, and the names of methods are labelled above their respective graphs. The light green area represents the 95% confidence interval. The PCC for each cell type can be found in Table S3.

**Figure S8 Deconvolution results for human_PBMC datasets**

This figure corresponds to NL_0 in the Figure S6, which means that noise is not added in this test. The datasets are annotated on the right side of the graph, and the names of methods are labelled above their respective graphs. The light green area represents the 95% confidence interval. The PCC for each cell type can be found in Table S3.

**Figure S9 Impact of rare component on the deconvolution results using coarse_bulk and mouse_tissue datasets**

**A.** The results for methods employing bulk data as a reference. **B.** The results for methods employing single-cell data as a reference. All component as well as rare component are illustrated respectively.

**Figure S10 Proportions predicted by different methods for UUO model**

The methods shown in the image are arranged in the same order as indicated in the legend on the right. sham, sham-operated; 2-day, 2-day post-ligation; 8-day, 8-day post-ligation.

**Table S1 Summary of the dataset used in Deconer**

**Table S2 Summary of the RNA-seq datasets**

**Table S3 Per cell type PCC results**
