# Supplementary material for: Deconer: An Evaluation Toolkit for Reference-based Deconvolution Methods Using Gene Expression Data
Source: Genomics Proteomics Bioinformatics. 2025 Feb 18;23(1):qzaf009. doi: 10.1093/gpbjnl/qzaf009 (PMC12221868; doi:10.1093/gpbjnl/qzaf009)
Supplement: qzaf009_Supplementary_Data [file qzaf009_supplementary_data.zip › Table S1.docx]

**Table S1 Summary of the dataset used in Deconer**

| **No.** | **Dataset name** | **Data type** | **Reference type** | **Proportion** | **Sample size** | **Ref.** |
| --- | --- | --- | --- | --- | --- | --- |
| 1 | Abbas | Microarray | Bulk | Known | 12 | [31] |
| 2 | Becht | Microarray | Bulk | Known | 10 | [6] |
| 3 | Gong | Microarray | Bulk | Known | 9 | [32] |
| 4 | Kuhn | Microarray | Bulk | Known | 10 | [33] |
| 5 | Linsley | RNA-seq | Bulk | Known | 5 | [34] |
| 6 | Liu | RNA-seq | Bulk | Known | 24 | [35] |
| 7 | Parsons | RNA-seq | Bulk | Known | 30 | [36] |
| 8 | Shen-Orr | Microarray | Bulk | Known | 33 | [37] |
| 9 | Shi | Microarray | Bulk | Known | 60 | [38] |
| 10 | T2D | RNA-seq | Single cell | Unknown | 89 | [39] |
| 11 | TCGA_LUSC | RNA-seq | Bulk | Unknown | 130 | [40] |
| 12 | TCGA_OV | RNA-seq | Bulk | Unknown | 514 | [40] |
| 13 | kidney_Arvaniti | RNA-seq | Single cell | Unknown | 11 | [41] |
| 14 | kidney_Arvaniti_TPM | RNA-seq | Bulk | Unknown | 11 | [41] |
| 15 | kidney_Craciun | RNA-seq | Single cell | Unknown | 19 | [42] |
| 16 | kidney_Craciun_TPM | RNA-seq | Bulk | Unknown | 19 | [42] |
| 17 | TCGA 35 cancer datasets | RNA-seq | Bulk | Unknown | - | [40] |

*Note*: All the datasets can be downloaded from [Deconer’s dataset page](https://honchkrow.github.io/Deconer_dataset/). Deconer, Deconvolution Evaluator.
