# Supplementary figures and images for: Deconer: An Evaluation Toolkit for Reference-based Deconvolution Methods Using Gene Expression Data
Source: Genomics Proteomics Bioinformatics. 2025 Feb 18;23(1):qzaf009. doi: 10.1093/gpbjnl/qzaf009 (PMC12221868; doi:10.1093/gpbjnl/qzaf009)

A

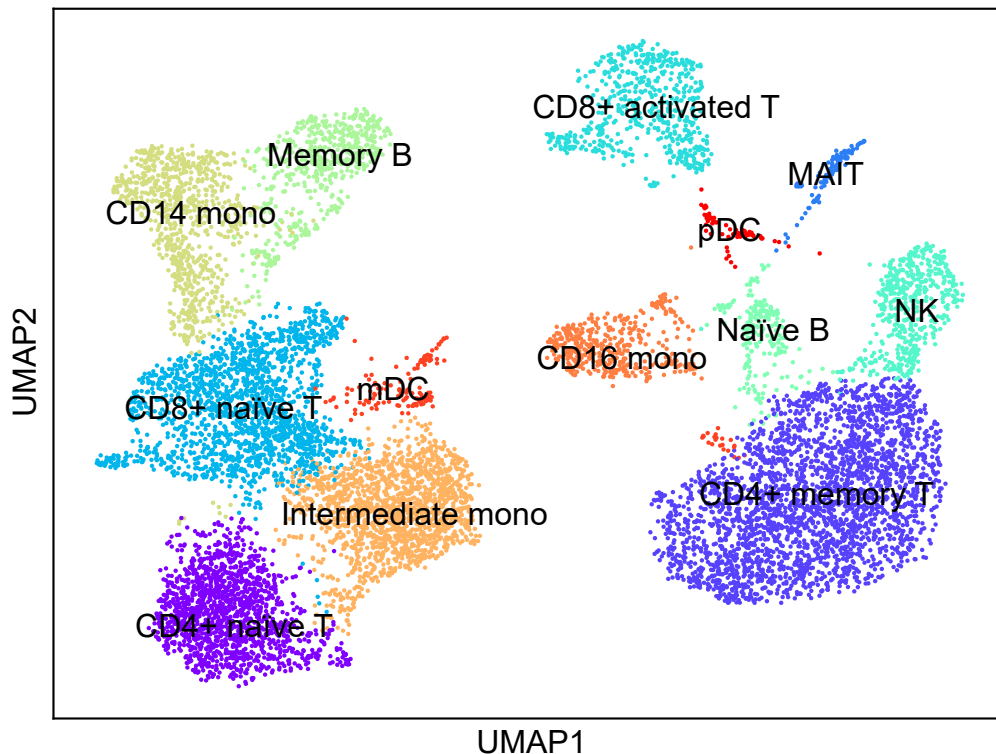

B

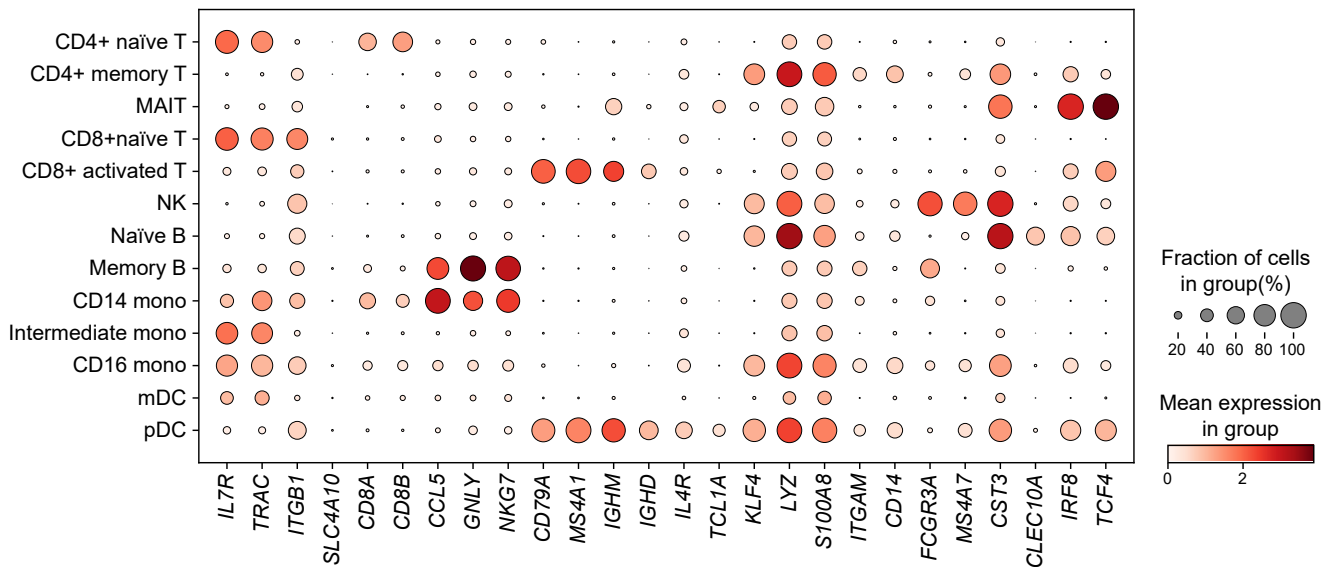

Supplement: qzaf009_Supplementary_Data [file qzaf009_supplementary_data.zip › Figure S1.pdf]

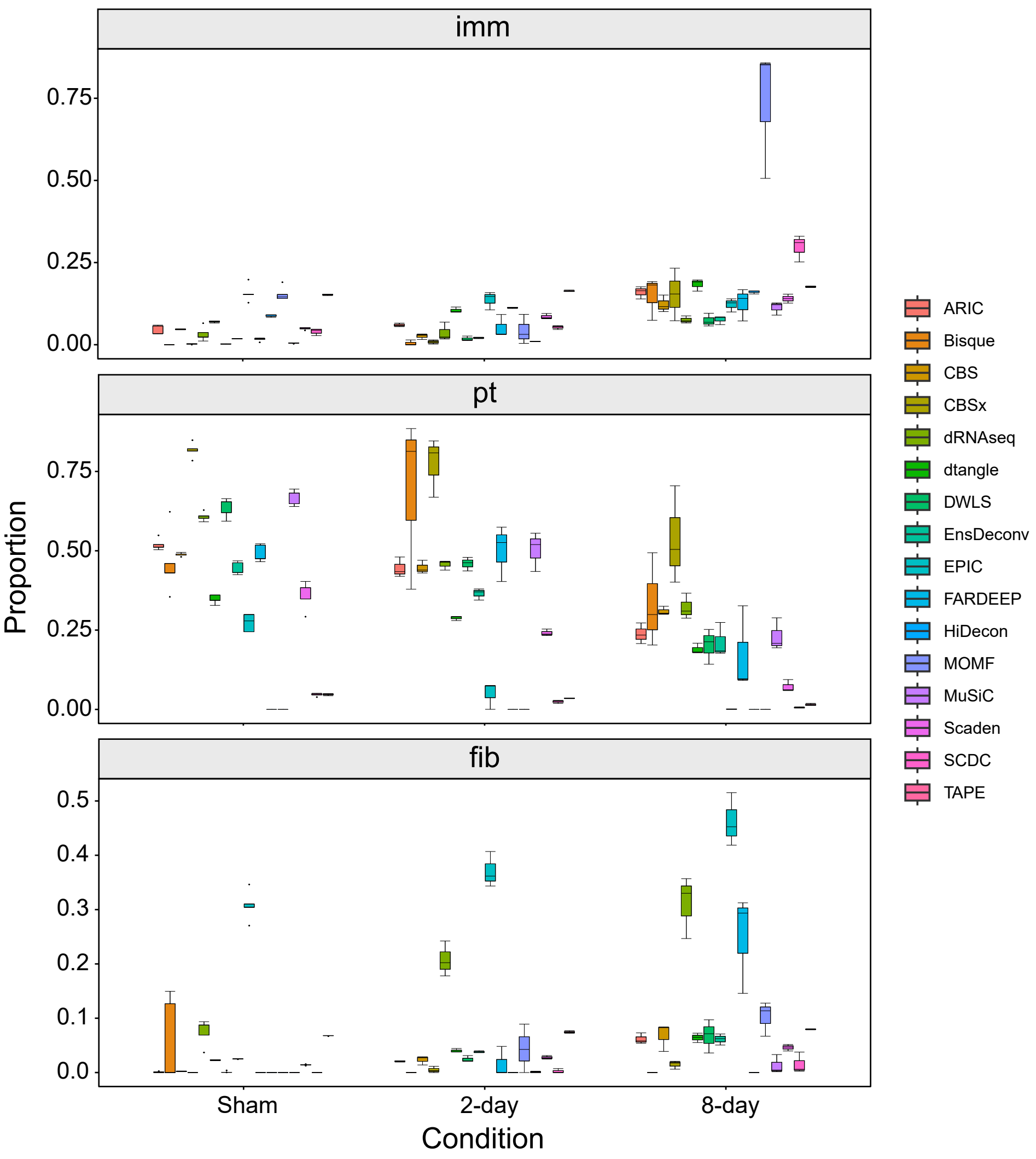

Supplement: qzaf009_Supplementary_Data [file qzaf009_supplementary_data.zip › Figure S10.pdf]

A

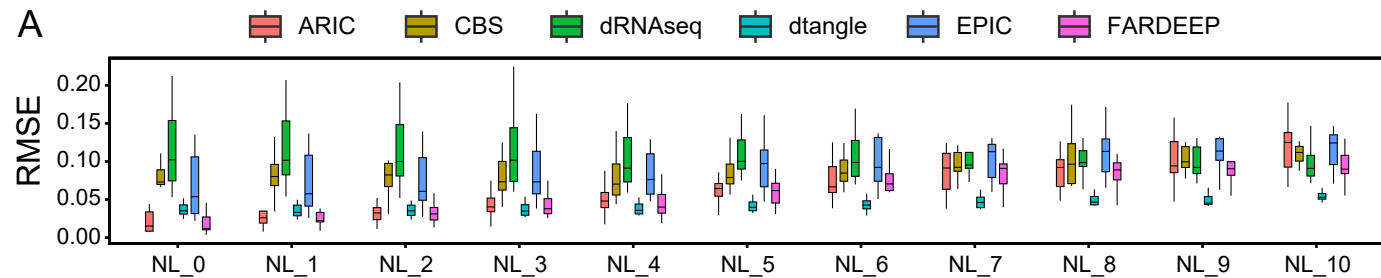

B

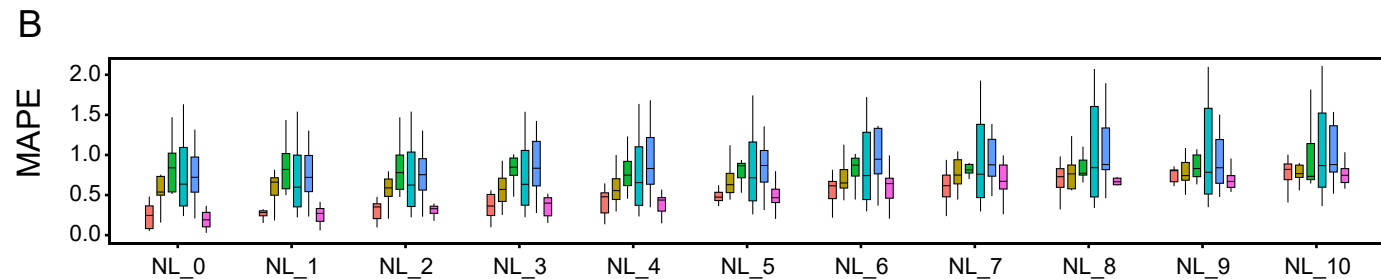

C

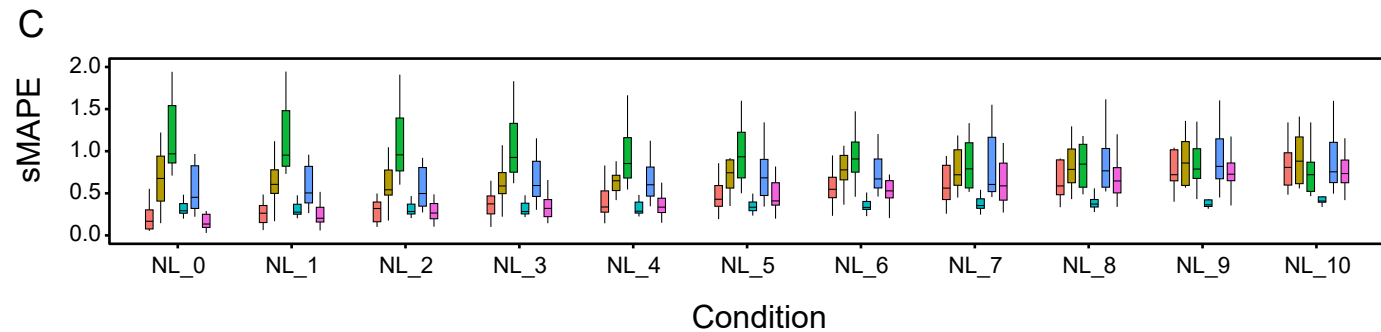

Supplement: qzaf009_Supplementary_Data [file qzaf009_supplementary_data.zip › Figure S2.pdf]

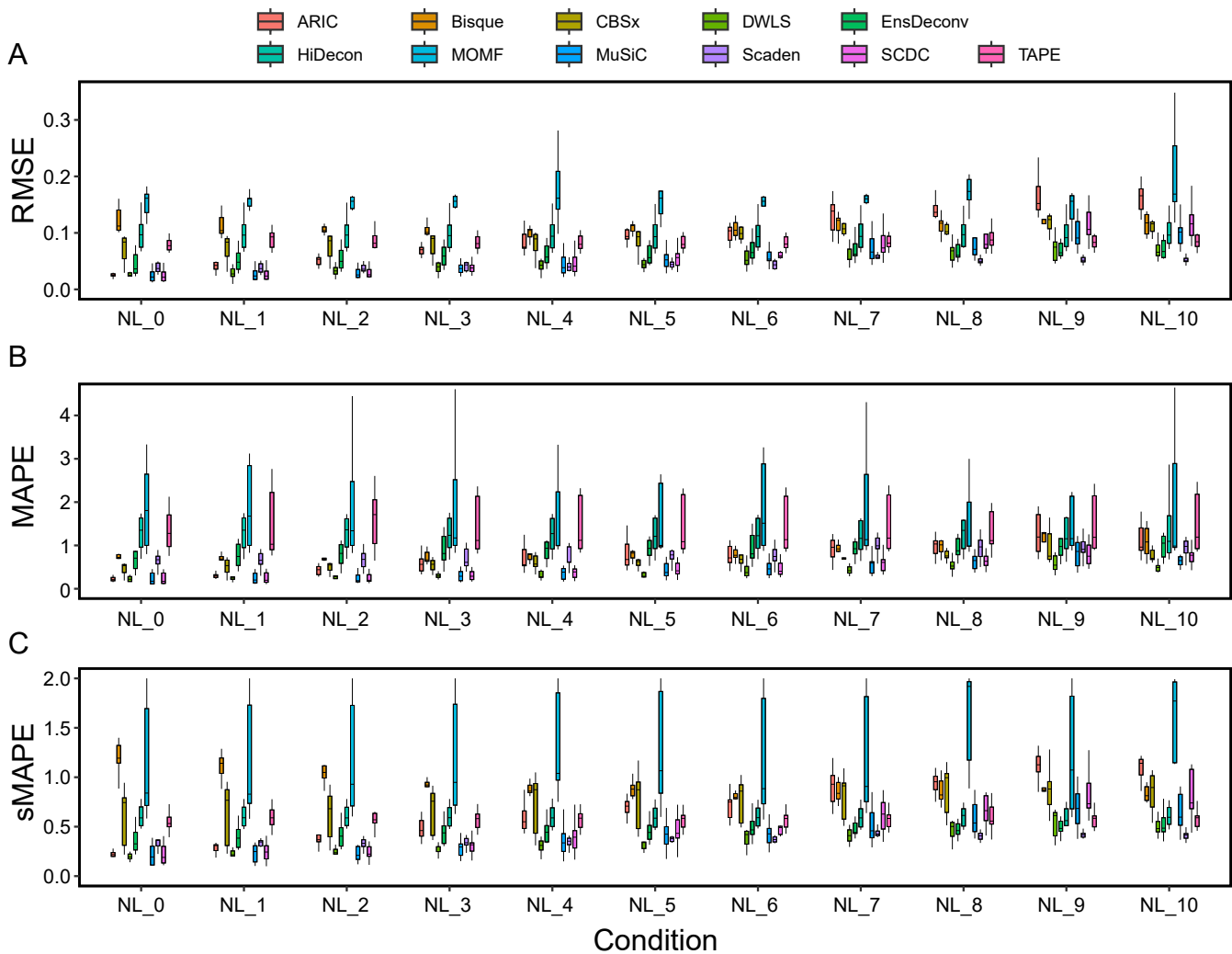

Supplement: qzaf009_Supplementary_Data [file qzaf009_supplementary_data.zip › Figure S3.pdf]

A

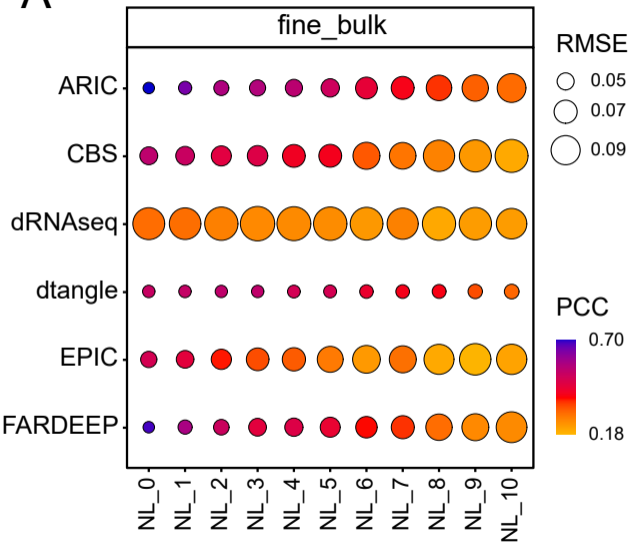

B

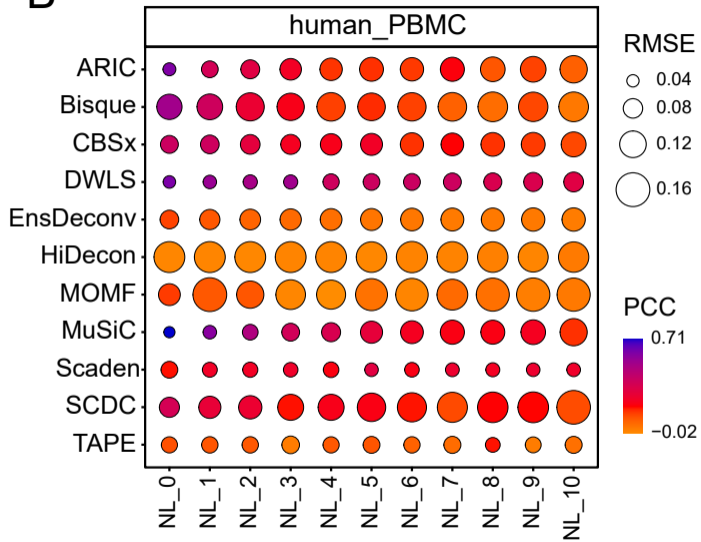

Supplement: qzaf009_Supplementary_Data [file qzaf009_supplementary_data.zip › Figure S4.pdf]

A

ARIC CBS dRNAseq dtangle EPIC FARDEEP

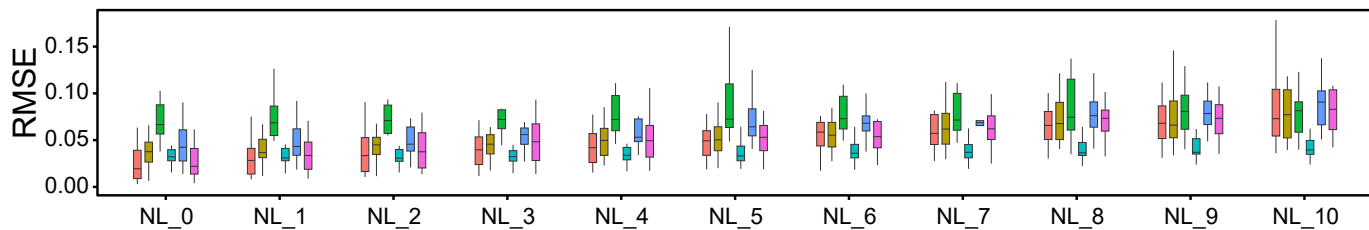

B

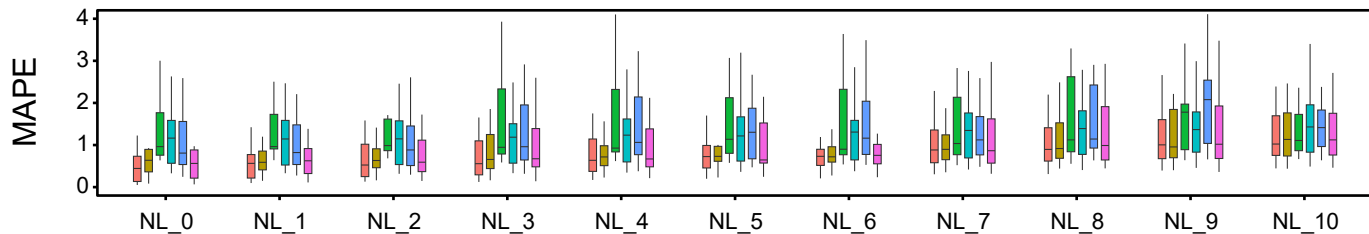

C

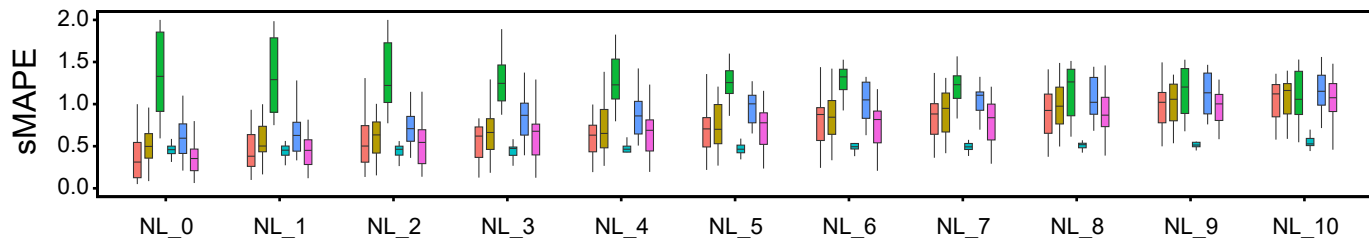

Condition

Supplement: qzaf009_Supplementary_Data [file qzaf009_supplementary_data.zip › Figure S5.pdf]

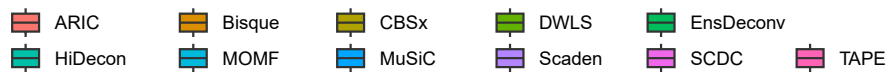

A

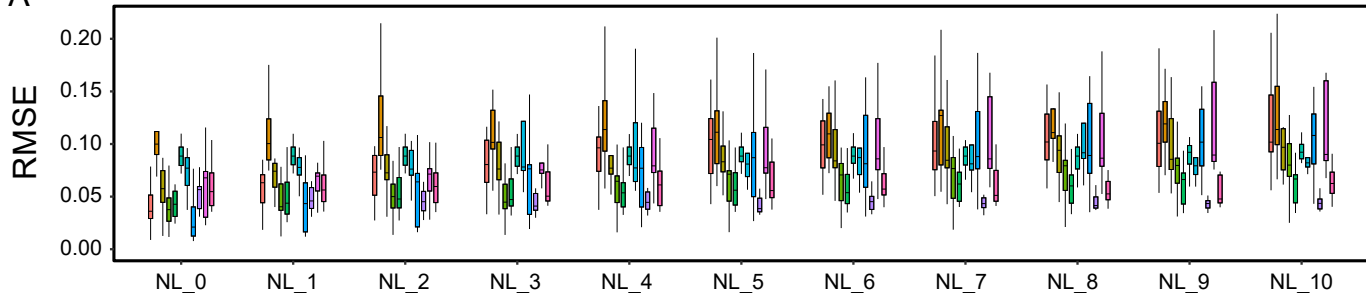

B

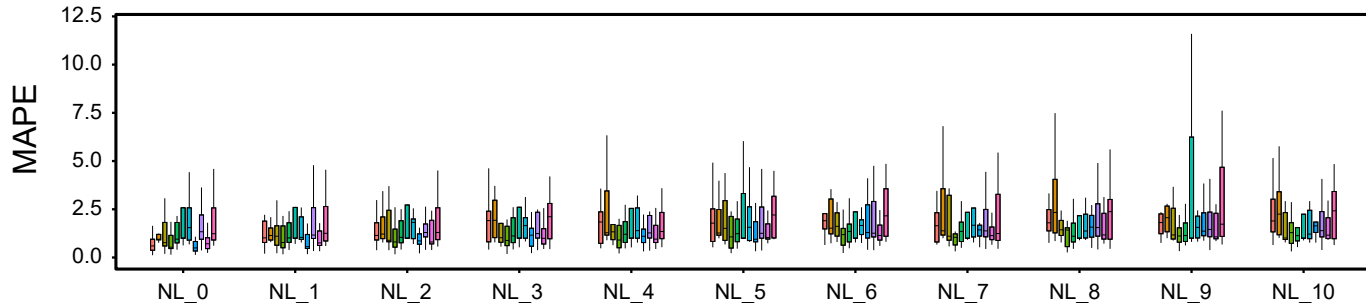

C

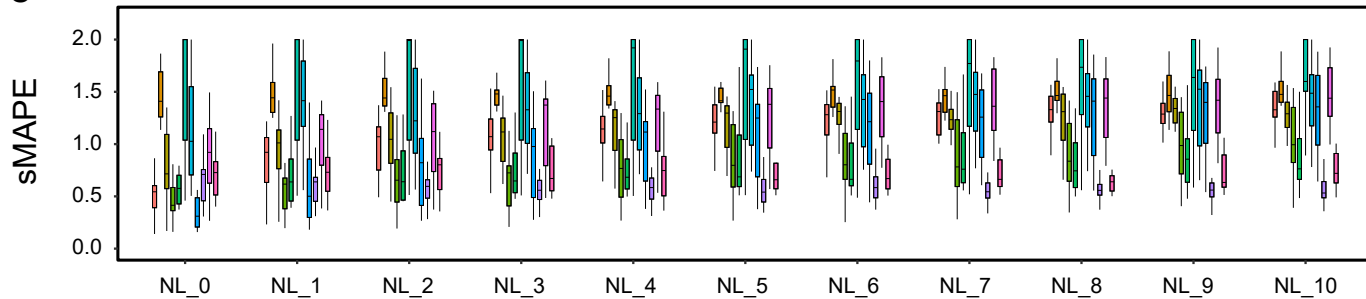

Supplement: qzaf009_Supplementary_Data [file qzaf009_supplementary_data.zip › Figure S6.pdf]

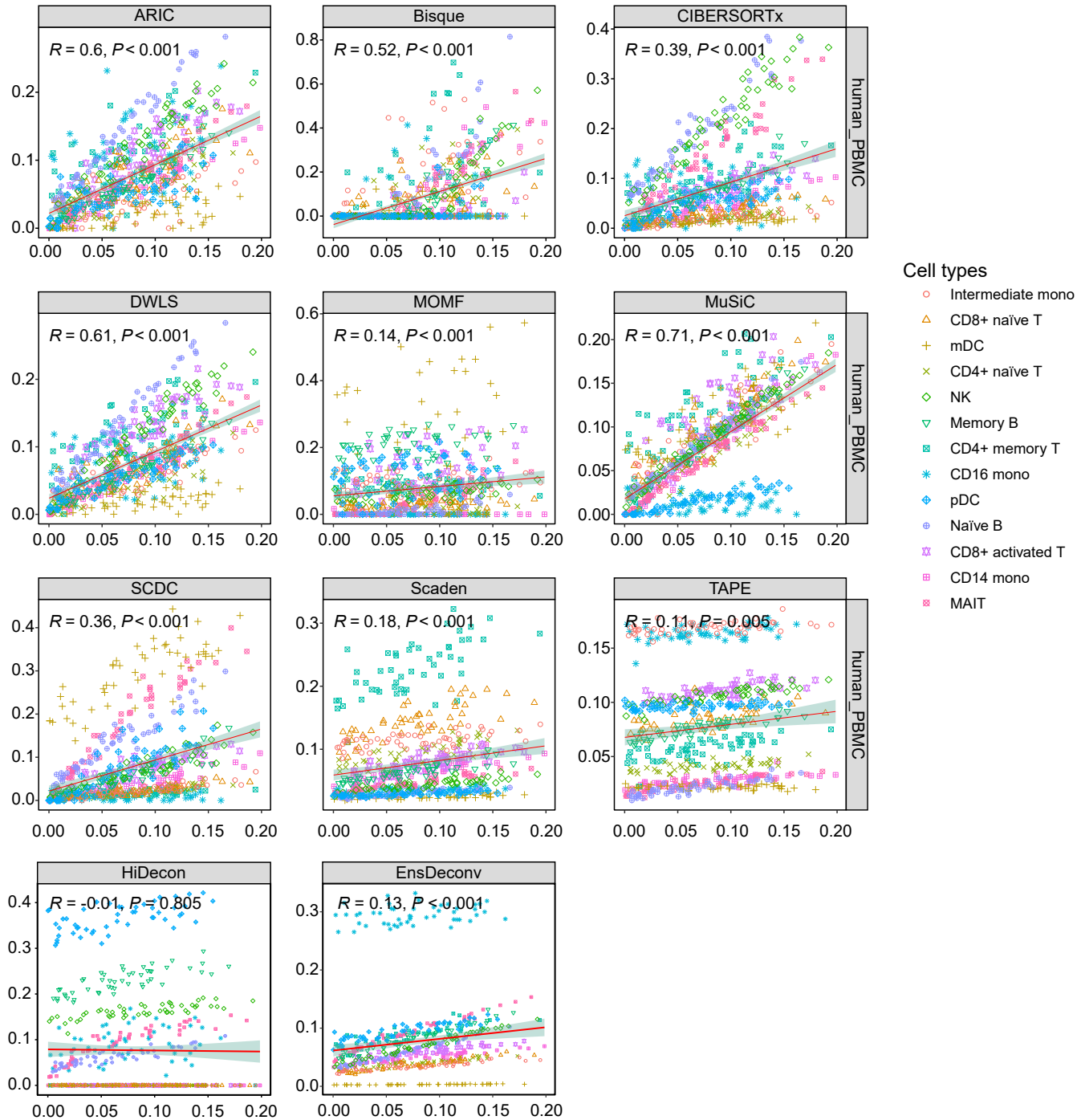

Supplement: qzaf009_Supplementary_Data [file qzaf009_supplementary_data.zip › Figure S8.pdf]
